# Maternal Plasma Proteins Associated with Birth Weight: A Longitudinal, Large Scale Proteomic Study

**DOI:** 10.1021/acs.jproteome.4c00940

**Published:** 2025-05-05

**Authors:** Ina Jungersen Andresen, Ane Cecilie Westerberg, Marie Cecilie Paasche Roland, Manuela Zucknick, Trond Melbye Michelsen

**Affiliations:** † Department of Obstetrics, Division of Obstetrics and Gynecology, 155272 Oslo University Hospital, 0372 Oslo, Norway; ‡ School of Health Sciences, Kristiania University College, Oslo 0107, Norway; § Department of Medical Biochemistry, Oslo University Hospital, 0450 Oslo, Norway; ∥ Department of Biostatistics, Oslo Centre for Biostatistics and Epidemiology, 6305 University of Oslo, 0372 Oslo, Norway; ⊥ Institute of Clinical Medicine, Faculty of Medicine, University of Oslo, 0372 Oslo, Norway

**Keywords:** Biomarkers, Fetal growth, Large for gestational age, Longitudinal, Pregnancy, Proteomics, Small for gestational age, SomaLogic

## Abstract

Small infants for gestational age (SGA) and large infants for gestational age (LGA) have increased risk of complications during delivery and later in life. Prediction of the fetal weight is currently limited to biometric parameters obtained by ultrasound scans that can be imprecise. Biomarkers of fetal growth would be crucial for tailoring clinical management and optimizing outcomes for the mother and child. Seventy pregnant women participated in the current study, including 58, 7, and 5 giving birth to adequate for gestational age (AGA), SGA, and LGA infants, respectively. Maternal venous blood was drawn at gestational weeks 12–19, 21–27, and 28–34 and quantified for nearly 5000 proteins on the SomaLogic platform. We used machine learning algorithms with leave-one-out cross-validation to construct multiprotein models for prediction of birth weight groups. Random forest models using only 20 predefined proteins (selected by moderated *t* tests) were able to predict LGA with good discrimination (AUC > 0.8) at all three visits, while prediction of SGA was less successful. Protein differential abundance analysis revealed 148 proteins with higher abundance in LGA compared to AGA pregnancies, while only four proteins were differentially abundant between the SGA and AGA. The principal findings indicate that the maternal plasma proteome may hold potential biomarkers of LGA.

## Graphical abstract

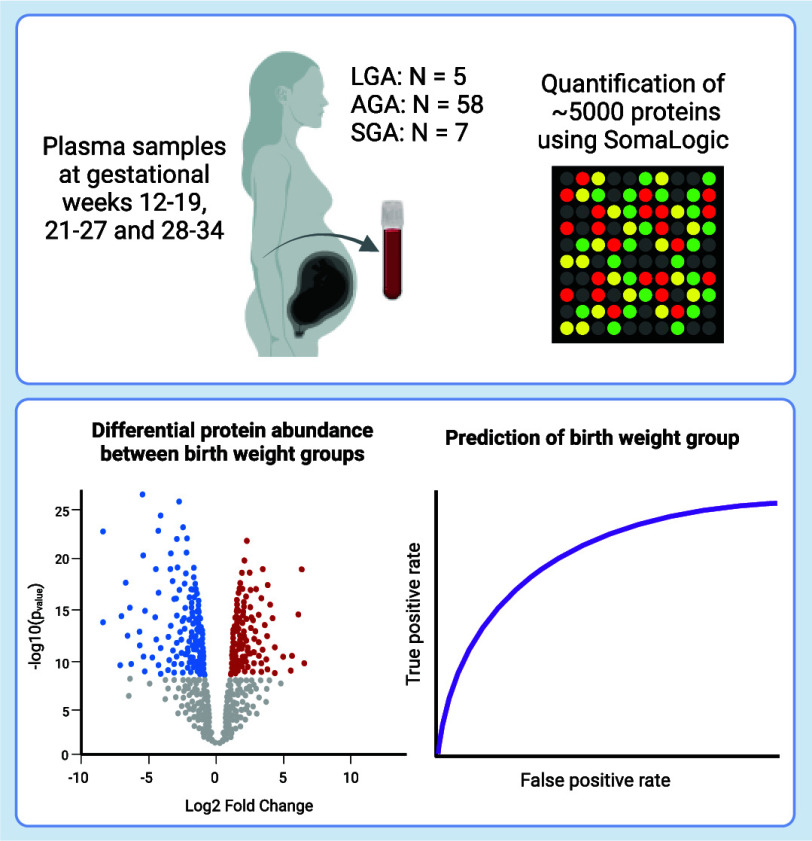

## Background

Adequate fetal growth is linked to better short and long-term health for the offspring.^1^ In utero growth can be evaluated retrospectively based on birth weight according to gestational age and sex. Infants are commonly defined as “adequate for gestational age” (AGA) if their birth weights are between the 10th and 90th percentile on the reference growth curves, whereas “small for gestational age” (SGA) and “large for gestational age” (LGA) infants have birth weights <10th and >90th percentiles, respectively. Mothers giving birth to SGA and LGA infants have increased need for interventions such as operative vaginal delivery and cesarean section,^2,3^ and SGA and LGA infants have increased neonatal morbidity, including need for neonatal intensive care.^4–6^ In the long term, both SGA and LGA infants are predisposed for obesity, hypertension, type 2 diabetes, as well as cardiovascular disease.^7–11^

Predicting the size of a baby before delivery could be beneficial for identification of risk pregnancies, considerations related to mode of delivery, and mitigation of risks associated with operative delivery. Identifying biomarkers associated with fetal size and growth trajectories across gestation would also represent an advancement as these proteins could reflect the general well-being of the fetus and the function and efficiency of the placenta. In-utero fetal weight estimation is traditionally performed using sonographic biometric measurements of the fetal biparietal diameter, head circumference, abdominal circumference, and femur length. Using these parameters, fetal weight can be estimated by formulas such as Hadlock’s^12,13^ or Combs’ formula.^14^ However, as ultrasound measurements can be imprecise, sonographic based approaches have shown inaccurate results in terms of in-utero diagnosis and prediction of fetal growth abnormalities.^2,15^ Additional tools to assess fetal growth and diagnose fetal growth deviations, such as protein biomarkers and predictive models, would therefore represent a significant advancement. Several studies have combined maternal risk factors and/or ultrasound data with a few selected protein markers from the maternal circulation to predict or identify SGA^16–21^ and LGA^22,23^ babies with promising, but also various results. Furthermore, promising results have been shown for studies examining metabolites^24^ and cell-free RNA in maternal circulation,^25–28^ encouraging further exploration, and advancement to multimarker models and prospective studies.^29,30^

Several studies have used large-scale proteomics to predict preterm birth,^31^ preeclampsia,^32–35^ and other hypertensive disorders in pregnancy,^36^ but as far as we know, few attempts have been made to construct large-scale multiprotein models in unbiased search of predictive biomarkers of fetal growth. In this secondary analysis of an existing data set including samples collected longitudinally across healthy pregnancies with nearly 5000 quantified plasma proteins,^33^ we aimed to 1) identify and describe differences in the plasma proteome of mothers giving birth to SGA, AGA, and LGA infants, 2) identify proteins associated with birth weight (z-score), and 3) identify protein biomarkers for prediction of birth weight group.

## Materials and Methods

### Study Design and Population

The study was based on a secondary analysis of a subsample of 70 participants originally designed as a preeclampsia prediction study.^33^ Exclusion criteria were multiple pregnancies, known pregestational diabetes, and severe chronic disease (lung, cardiac, gastrointestinal, or renal). Fasting antecubital vein plasma samples were obtained at three visits across gestational weeks; 12–19, 21–27, and 28–34. Participants were recruited at Oslo University Hospital, Rikshospitalet between 2002 and 2008 and signed a written informed consent upon participation. The study was approved by the Regional Committee for Medical and Health Research Ethics, Southern Norway, Oslo, Norway (reference number S-2014/224-0119a).

### Calculation of z-Scores

Birth weight z-scores and birth weight groups (SGA, AGA, and LGA) were calculated according to Norwegian references adjusted for sex and gestational age.^37^ Infants with birth weight z-score >1.3 (>90th percentile) were defined as LGA, and infants with birth weight z-scores < -1.3 (<10th percentile) were defined as SGA.

### Measurements of Protein Abundance and Data Processing

Protein abundance was measured using the microarray-based proteomic platform SomaScan assay version 4.0, which can quantify 4979 proteins simultaneously, and reports protein quantity in relative fluorescent units (RFUs) proportional to protein concentration.^38^ Proteins are referred to by gene names provided by the HUGO gene nomenclature committee. Of the 4979 measured proteins, 4565 passed quality filtration and were continued for downstream analysis.

To limit the effect of extreme outliers, RFU values >2*98th quantile were moved to the 2*98th quantile for each respective protein (data winsorization). The data were further log2-transformed and detrended by calculating protein Multiples of the Mean (MoM) to account for variability in gestational age within the three visits. First, using the *mgcv* R-package (v. 1.9-1),^39^ generalized additive models were modeled across gestation for each protein using the AGA samples only. Different smoothing parameters were tested (knots = 3, 4, 5, and 6) and the best parameter (based on r-squared) were selected for each protein. MoM values of all protein values were then calculated by subtracting the modeled value from the respective log2 RFU value.

Samples were clustered on MoM-values within each visit, and visualized by principal component analysis (PCA) using the *pcaMethods* (v. 1.86.0) package in R.^40^

### Statistical Analysis of Clinical Variables

Clinical variables were normally distributed () and presented as mean values with standard deviation for continuous variables and absolute values and proportions for categorical variables according to SGA, AGA, and LGA groups. Differences between groups were compared by Welch’s two sample *t* tests for continuous variables and Fisher’s exact tests for categorical variables.

Spearman correlation between birth weight z-scores and continuous clinical variables were calculated using the *psych* (v. 2.2.9) package in R,^41^ and Welch’s two samples *t* tests were used for comparing birth weight z-scores according to binary variables. Significance was inferred by two-sided p-values < 0.05.

### Prediction of Birth Weight Groups and Evaluation of Biomarker Signatures

To investigate whether the plasma proteome can be used to predict birth weight, we constructed binary elastic net (EN) and random forest (RF) multiprotein models for prediction of birth weight group (LGA vs AGA and SGA vs AGA) at each visit using the *tidymodels* (v. 1.1.1)^42^ and *randomForest* (v. 4.7-1.1)^43^ R-packages. Both algorithms are popular for analyzing high dimensional data, often used as benchmarking methods due to their consistently good performance, especially in settings with a relatively small sample size. Both methods often achieve good prediction performance in independent validation data due to their ability to reduce overfitting by regularization through penalization (EN)^44^ and bootstrapped sampling and averaged predictions (RF).^45^ The methods are complementary, since while EN assumes linear effects, RF is particularly good at detecting nonlinear and interaction effects. Both methods perform feature selection, by penalized regression (EN) or importance ranking (RF), giving the combination of proteins with the best predictive value for the outcome variable that has the most impact in the model. The models were trained by leave-one-out cross-validation (LOO-CV) to limit the effect of overfitting. In addition to models selecting among all measured proteins, we constructed reduced models selecting among 50, 40, 30, 20, 10, and 5 proteins selected by p-value ranking from moderated *t* tests at each CV iteration. For the elastic net models, the alpha (α) and lambda (λ) hyperparameters were tuned by 10*10 different α and λ combinations using the *tune* (v. 1.1.2) R-package.^45,46^ For the random forest models, the number of trees was set to 500, and otherwise default parameters were used. Model fit was evaluated by area under the receiver operating characteristic curve (AUC ROC) and plotted using the pROC (v. 1.18.5) R-package.^47^ Due to the imbalance between cases and controls, F1-score, balanced accuracy, and area under the precision-recall curve (AUC-PR) were calculated for each of the final models to evaluate the predictive outcome of the cases.

### Differential Protein Abundance between Birth Weight Groups

Differential protein abundances between groups were identified by moderated *t* tests using the *limma* (v. 3.50.0) package in R^48^ while adjusting for BMI and nulliparity. The p-values were adjusted for multiple testing by controlling the false discovery rate.^49^ Proteins with adjusted p-values (q-values) < 0.05 were considered as statistically significant.

### Linear Regression between Protein Abundance and Birth Weight

Linear regression models with *limma* (v. 3.50.0) were used to investigate the association between protein abundance for each of the proteins and birth weight z-score while adjusting for nulliparity and BMI. The p-values were adjusted for multiple testing by controlling the false discovery rate.^49^ Proteins with q-values < 0.05 were considered as statistically significant.

### Functional Annotation by Over-Representation Analysis

Over-representation analysis (ORA) of Biological process gene ontologies (GOs) was performed on proteins with differential abundance between groups (SGA, AGA, and LGA), and proteins correlated with birth weight z-scores using the *clusterProfiler* (v. 4.12.6) package in R.^50^ All measured proteins passing quality filtration (4565) were used as the background. The p-values were adjusted for multiple testing by controlling the false discovery rate, and ontologies with q-values < 0.2 and a minimum of three proteins mapping to the respective ontology/pathway were considered as significantly over-represented. The simplify function was used to concatenate similar ontologies. The results were visualized in hierarchical tree plots using the *enrichplot* (v. 1.24.4) package in R.^51^

### Modeling of Longitudinal Protein Abundances

Generalized adaptive models were fitted to each birth weight group using the *mgcv* (v. 1.9-1) R-package^39^ to display mean longitudinal trajectories of protein abundances weight group. Number of knots (k = 3, 4, 5, or 6) for each protein model were selected based on the highest r-squared value.

## Results and Discussion

### Demographics and Group Comparisons

A total of 70 women participated in the study, including 7, 58, and 5 women who gave birth to SGA, AGA, and LGA infants, respectively (Table 1). Mothers of SGA infants had a significantly lower placental weight than mothers of AGA infants. There were otherwise no differences in maternal factors between the groups.

**Table 1:** Clinical Variables of the Study Population

	SGA (*n* = 7)	AGA (*n* = 58)	LGA (*n* = 5)	p-value^a^
**Maternal parameters**				
Age (years), mean (SD)	33.0 (1.5)	32.0 (3.9)	32.6 (1.7)	0.20^b^, 0.51^c^, 0.68^d^
Height (cm), mean (SD)	165 (5.8)	169 (6.4)	172 (6.7)	0.14^b^, 0.30^c^, 0.08^d^
Weight (kg), mean (SD)^e^	64.5 (11.0)	68.6 (8.9)	73.6 (12.3)	0.38^b^, 0.41^c^, 0.22^d^
BMI (kg/m2), mean (SD)^e^	23.8 (3.6)	24.1 (2.8)	24.8 (4.0)	0.80^b^, 0.72^c^, 0.65^d^
Gestational weight gain (kg), mean (SD)^f^	10.1 (4.1)	9.9 (3.8)	12.7 (3.7)	0.93^b^, 0.17^c^, 0.27^d^
Nulliparous/Multiparous, N (%)	5/2 (71/29)	29/29 (50/50)	1/4 (20/80)	0.43^b^, 0.36^c^, 0.24^d^
Smoking during pregnancy (No/Quit during pregnancy/Yes), N (%)	4/3/0 (57/43/0)	45/12/1 (77/21/2)	5/0/0 (100/0/0)	0.41^b^, 0.61^c^, 0.20^d^
Higher education (Yes/No), N (%)^g^	4/3 (57/43)	50/8 (86/14)	4/1 (80/20)	0.09^b^, 0.55^c^, 0.58^d^
Marital status (Partner/Single), N (%)	7/0 (100/0)	58/0 (100/0)	5/0 (100/0)	1.0^b^ ^c^ ^d^
Gestational age visit 1, mean (SD)	15.2 (1.1)	15.7 (1.2)	15.9 (0.52)	0.23^b^, 0.53^c^, 0.14^d^
Gestational age visit 2, mean (SD)	23.2 (0.92)	23.4 (1.0)	23.4 (1.3)	0.47^b^, 0.91^c^, 0.77^d^
Gestational age visit 3, mean (SD)	31.5 (0.87)	31.3 (0.97)	30.8 (0.89)	0.54^b^, 0.31^c^, 0.21^d^
**Neonatal parameters**				
Birth weight (g), mean (SD)	2776 (243)	3567 (328)	4430 (242)	<0.001^b^ ^c^ ^d^
Birth weight (z-score), mean (SD)	–1.9 (0.35)	–0.12 (0.58)	1.7 (0.30)	<0.001^b^ ^c^ ^d^
Placental weight (g), mean (SD)	557 (58.2)	710 (142)	787 (235)	<0.001^b^, 0.63^c^, 0.23^d^
Birth weight (g)/Placental weight (g), mean (SD)	4.9 (0.51)	5.2 (0.82)	6.3 (2.4)	0.35^b^, 0.51^c^, 0.44^d^
Fetal length (cm), mean (SD)	47.9 (2.6)	51.2 (1.9)	54.1 (2.2)	0.03^b^,0.03^c^, 0.002^d^
Sex (Female/Male), N (%)	4/3 (57/43)	25/33 (43/57)	1/4 (20/80)	0.69^b^, 0.39^c^, 0.29^d^

a The p-values obtained by Welch’s two sample *t* tests for numerical variables and fisher’s exact test for categorical variables.

b SGA versus AGA

c LGA versus AGA.

d SGA versus LGA.

e Based on self-reported weight at visit 1 (week 12–19).

f Weight gain from visit 1 (week 12–19) to visit 4 (week 34–38).

g University/University college degree.

### Correlation between Clinical Variables and Birth Weight

Birth weight z-scores were higher in multiparous compared to nulliparous participants (p = 0.04) (Figure 1C). No significant association was found between birth weight z-score and maternal BMI, age, or fetal sex (Figure 1A,B,D).

**Figure 1:**
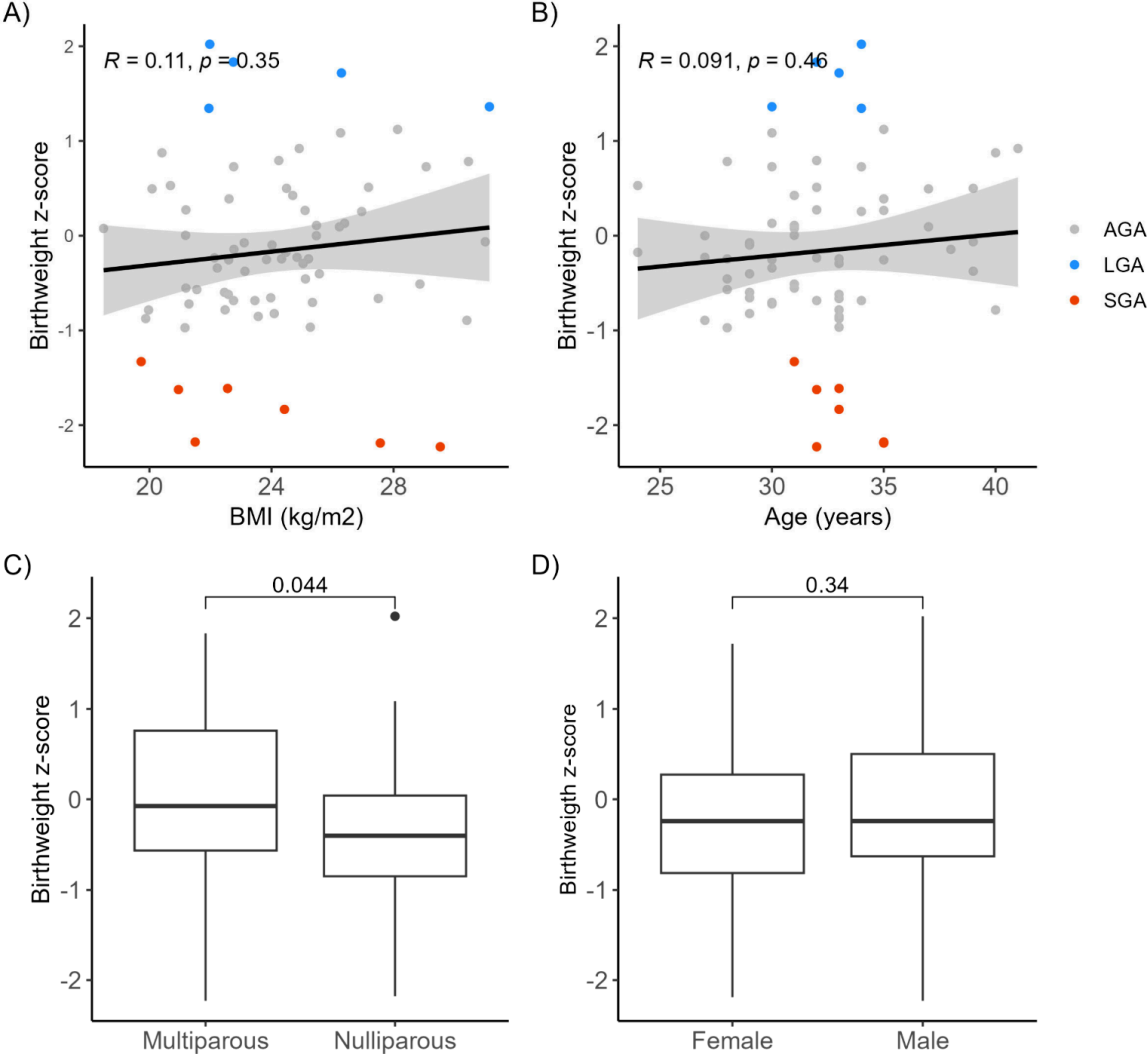
Pearson correlation between birth weight z-score and BMI (A) and maternal age (B). Welch’s two samples *t* test between birth weight z-score and nulliparity (C), and fetal gender (D).

### Principal Component Analysis of Proteins in Relation to Birth Weight Group

Principal component analyses of the 4565 proteins did not place the SGA or LGA pregnancies in any distinct cluster at either visit 1, 2, or 3 (Figure 2A, B and C). Nevertheless, there was a tendency toward separation of SGA and LGA samples along the second principal component at visit 1 and 3, and the first principal component at visit 2 (visual inspection).

**Figure 2:**
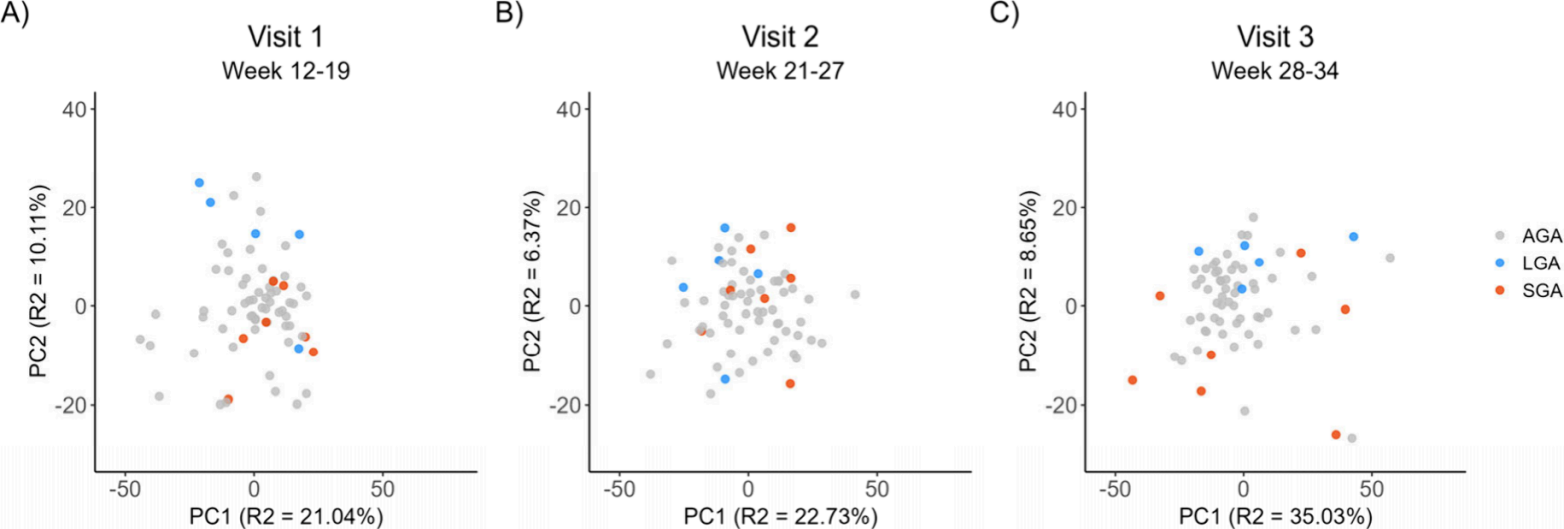
Principal component analysis for visualization of sample clustering at visit 1 (A), visit 2 (B), and visit 3 (C). There are no apparent clusters at either of the visits, although SGA and LGA samples seem to separate along PC2 at visits 1 and 3. The samples at visit 2 were less scattered than those at visits 1 and 3.

### Prediction of Birth Weight Group

Two different machine learning algorithms (elastic net (EN) and random forest (RF)) were explored to predict the birth weight group using protein abundances. In general, sparser RF models tended to predict LGA with a higher accuracy than models with all 4565 proteins (). RF models with 20 proteins (selected by moderated *t* tests) predicted LGA with good accuracy^52^ at all three visits (visit 1 AUC = 0.85, visit 2 AUC = 0.86, and visit 3 AUC = 0.80), while low to moderate performance was achieved for the RF models selecting proteins among all 4565 proteins (visit 1 AUC = 0.65, visit 2 AUC = 0.78, and visit 3 AUC = 0.73) (Figure 3A). The sparse EN models also had higher performance than the models containing all proteins (). EN models with 20 proteins showed good performance at all three visits (visit 1 AUC = 0.78, visit 2 AUC = 0.86, and visit 3 AUC = 0.78), while models containing all proteins showed moderate performance (visit 1 AUC = 0.69, visit 2 AUC = 0.70, and visit 3 AUC = 0.71). These findings may indicate high variability in proteomic profiles among women giving birth to LGA infants, and the importance of selecting a few good biomarker candidates. Although the models displayed generally good performance, F1-scores ranging from 0 to 0.33, balanced accuracy of 0 for all models, and area under the precision-recall curve (AUC-PR) < 0.59 illustrate that the promising performance is mostly due to correct classification of AGAs, but incorrect classification of LGA cases.

**Figure 3:**
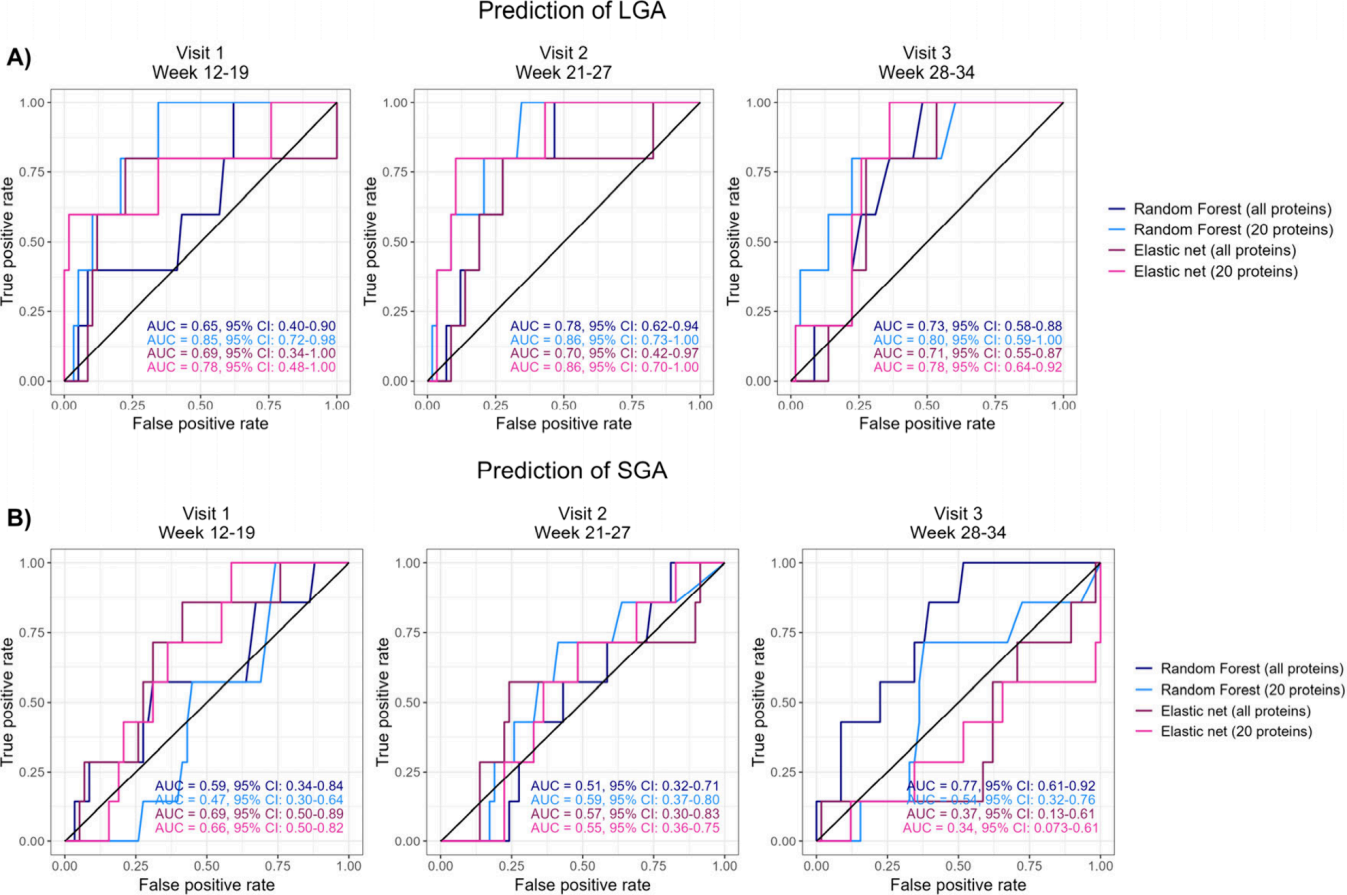
Receiver operating characteristic (ROC) curves for prediction of LGA (A) and SGA (B) by elastic net (EN) and random forest (RF) models at visits 1, 2, and 3. Results were obtained using leave-one-out cross-validation. Models with “all proteins” are constructed with all 4565 proteins, while models with 20 proteins are constructed with the top 20 proteins (ranked on p-values) from moderated *t* tests between groups at each cross-validation iteration.

Models predicting SGA had lower AUCs and larger 98% confidence intervals than the models predicting LGA (Figure 3B and ). The only acceptable model was RF with all proteins at visit 3 with an AUC of 0.77. However, also this model displayed poor ability to correctly classify SGA cases with F1 score = 0.25, balanced accuracy = 0, and AUC-PR = 0.11. The poor predictive performance may be a reflection of large individual differences in protein abundance among SGA mothers or composition between study objects (supported by the principal component analysis), and also a need for a larger study sample.

### Differential Proteomic Profiles between Birth Weight Groups

Differential abundance analysis identified 148 differential proteins between LGA and AGA pregnancies (q-value < 0.05) (Figure 3A–C and ). All of these 148 proteins had higher abundance in LGA samples compared to AGA, possibly indicating that several biological processes were upregulated in women carrying large babies. Sixty proteins had higher abundance in LGA pregnancies across all three visits, while 39, 12, and 9 proteins were differentially abundant at visit 1, 2, and 3 exclusively (Figure 4D). Although not significant at all three visits, all 148 proteins had constantly higher abundances in the LGA samples across gestation (Figure 4E). The p-value histograms from the LGA vs AGA tests support that the null hypothesis (i.e., that there is no difference between tested groups for any of the proteins) is false (). Over-representation analysis (ORA) showed that the 148 differential proteins were enriched for gene ontology biological processes associated with ossification and osteoblast differentiation, and the canonical and noncanonical Wnt signaling pathway (Figure 5), probably reflecting increased growth and development in LGA compared to AGA.

**Figure 4:**
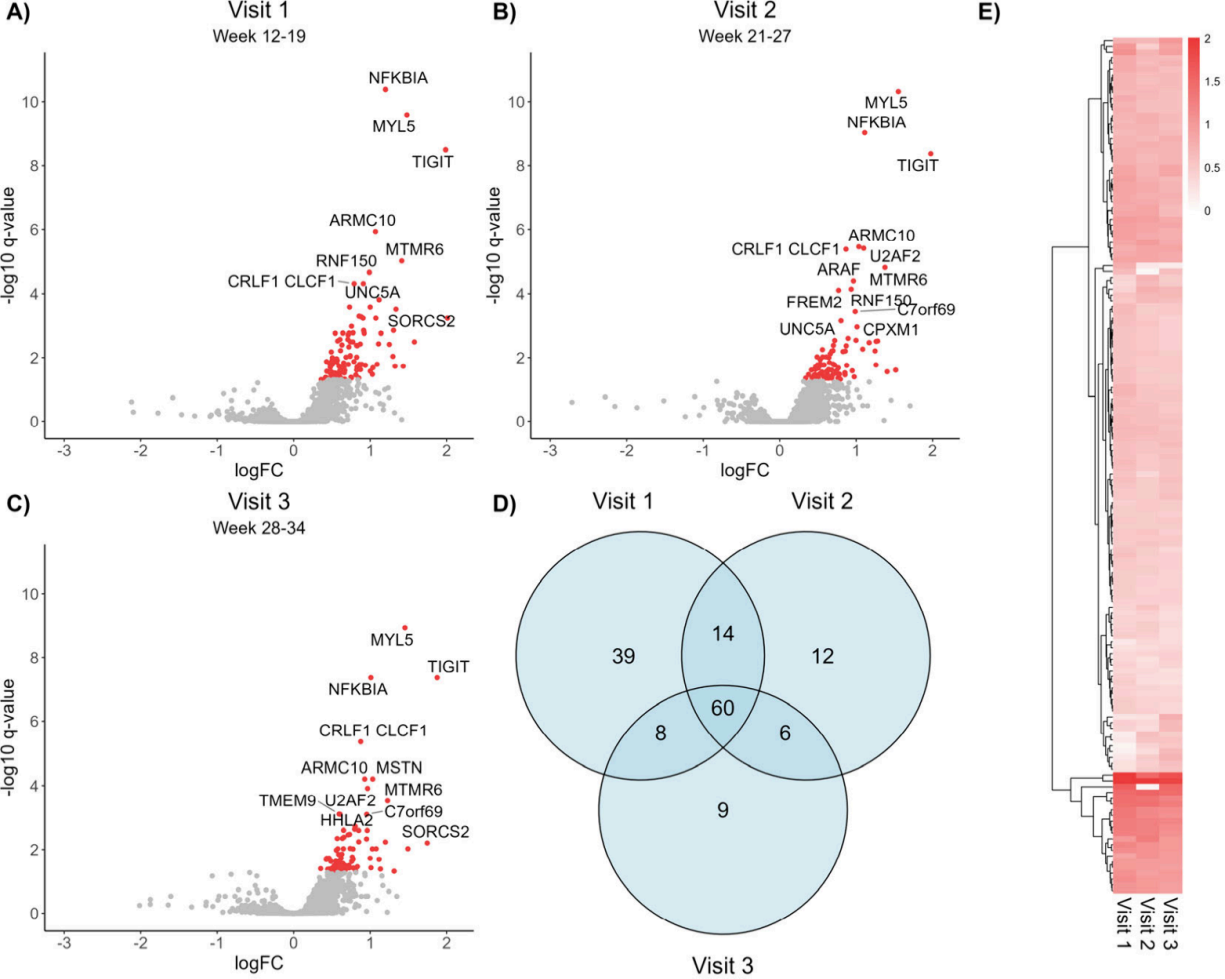
Volcano plots of differential abundance analysis between LGA and AGA groups at visit 1 (A), visit 2 (B), and visit 3 (C). The degree of difference (given by log2 fold change) is displayed by the *x*-axis, and significance level (given by -log10 q-value) is displayed along the *y*-axis. Positive log2 fold change means higher abundance in LGA compared to AGA, and negative log2 fold change means lower abundance in LGA. Red dots display proteins with significantly (q-value < 0.05) higher abundance in LGA compared to AGA. Nonsignificant proteins (q-value > 0.05) are annotated in gray. D) Venn diagram displaying the overlap of significantly differential proteins at the three visits. E) Heat map of Log2 fold change of the 148 significant proteins at visits 1, 2, and 3.

**Figure 5:**
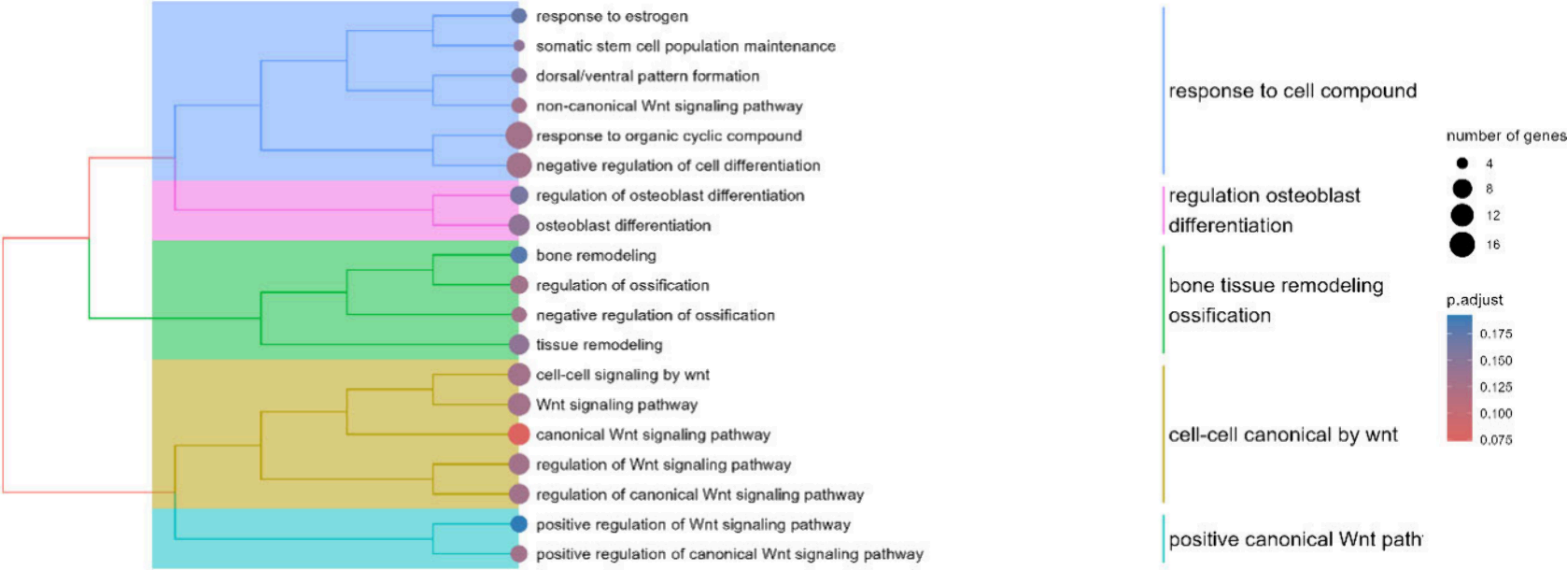
Tree plot of the significantly (q-value < 0.2) enriched biological processes gene ontologies among the 148 differential proteins between LGA and AGA. Ontologies are clustered by Jaccard’s similarity.

Four proteins were differentially abundant between the SGA and AGA pregnancies. C1orf210 and ITGA2 were significantly higher in SGA compared to AGA at all three visits, while NHEJ1 was significantly higher and TNC was significantly lower in SGA at visit 3 only (Figure 6A–D and ). The uniform distribution of p-value histograms from the SGA vs AGA tests at visit 2 and 3 () may suggest that the null hypothesis is true globally, but it also reflects a low statistical power of the differential abundance tests due to small sample sizes.^53^ At visit 1, the p-value histogram showed slightly overabundance of small p-values indicating differences in abundance between SGA and AGA samples, but insufficient statistical power. Other studies have identified proteomic differences in SGA vs AGA pregnancies, despite measuring less proteins and fewer participants.^54,55^ An explanation for our lack of significant results could be that the high number of measured proteins and thus tests, results in a strict correction for multiple testing.^56^ Inclusion of solely term pregnancies (>37 weeks) in the current analysis may also contribute to limited findings, as previous studies have illustrated that the biggest differences in biochemical markers between AGA and SGA cases has been driven by SGA with preterm delivery.^17–19,57^

**Figure 6:**
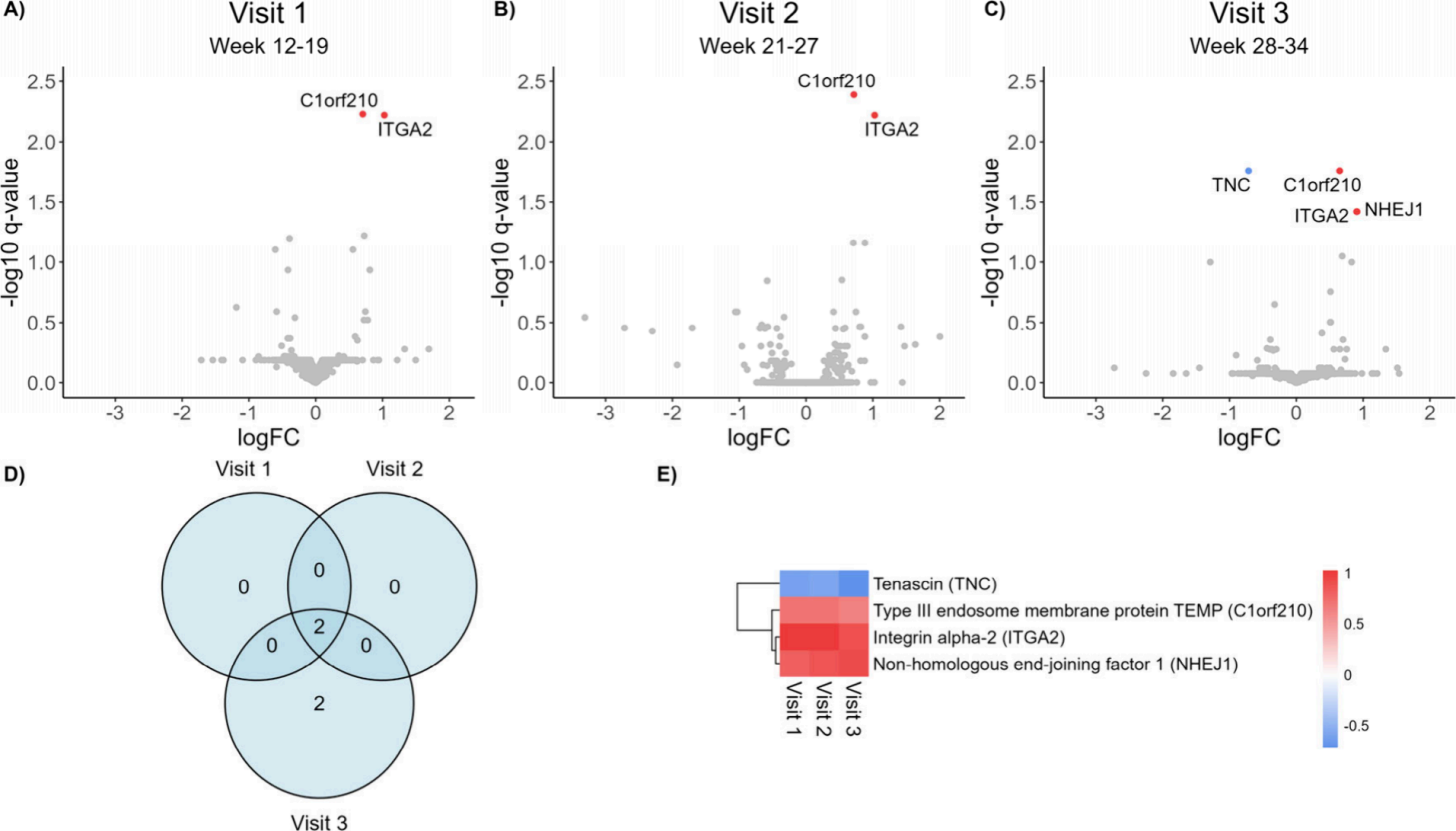
Volcano plots illustrating differential abundance analysis between SGA and AGA groups at visit 1 (A), visit 2 (B), and visit 3 (C). The degree of difference (given by log2 fold change) is displayed by the *x*-axis, and significance level (given by -log10 q-value) is displayed along the *y*-axis. Positive log2 fold change means higher abundance in SGA compared to AGA, and negative log2 fold change means lower abundance in SGA. Proteins with significantly (q-value < 0.05) higher abundance in SGA compared to AGA are marked in red and proteins with significantly lower abundance in SGA are marked in blue. Nonsignificant proteins (q-value > 0.05) are marked in gray. D) Venn diagram of the differential proteins at visits 1, 2, and 3. E) Heat map of log2 fold change of the significant proteins at visits 1, 2, and 3.

### Proteins Associated with Birth Weight z-Score

As our data set contains a rather small number of LGA and SGA cases, we also wanted to investigate proteins associated with birth weight z-scores. Linear regression, adjusted for nulliparity and BMI, showed that 550 proteins were significantly associated with the birth weight z-score, among which 96 proteins were significantly different at visit 1, zero proteins at visit 2, and 523 proteins at visit 3 (Figure 7A and ). Sixty-nine proteins were significantly associated with birth weight z-score at both visits 1 and 3. Overabundance of low p-values () supports the rejection of the null-hypothesis and indicates that the experiment was sufficiently powered. Twenty-eight proteins were significantly associated with birth weight z-score and differentially abundant between birth weight groups (Figure 7B). These 28 proteins were all positively correlated with birth weight z-score (Figure 7C). Although there was a higher number of significant proteins at visit 3, many proteins had a stronger correlation (determined by Spearman’s ρ) at visit 1 (Figure 7D), indicating a possibility to predict LGA pregnancies already in early pregnancy. Clinical advantages from such a prediction are several. First, it would allow for the implementation of maternal lifestyle and dietary interventions to manage conditions such as gestational diabetes, which can contribute to fetal overgrowth.^22,58^ This can help maintain healthy maternal weight and glucose levels in pregnancy, potentially reducing the likelihood of an LGA pregnancy. Second, identifying LGA fetuses in the first trimester would enable closer monitoring of fetal growth and development. Regular evaluations including ultrasound and noninvasive prenatal testing could be scheduled to ensure fetal well-being and timely identification of emerging complications. Third, early identification would provide ample time for obstetricians to plan for safe delivery. This could include induction of labor to avoid complications such as shoulder dystocia and birth trauma.^59,60^ Fourth, anticipating an LGA infant allows for the implementation of preventive measures to reduce neonatal complications, such as hypoglycemia and respiratory issues. Fifth, hospital resources can be allocated more efficiently to ensure that trained medical staff and equipment are available during labor and delivery to handle potential complications.

**Figure 7:**
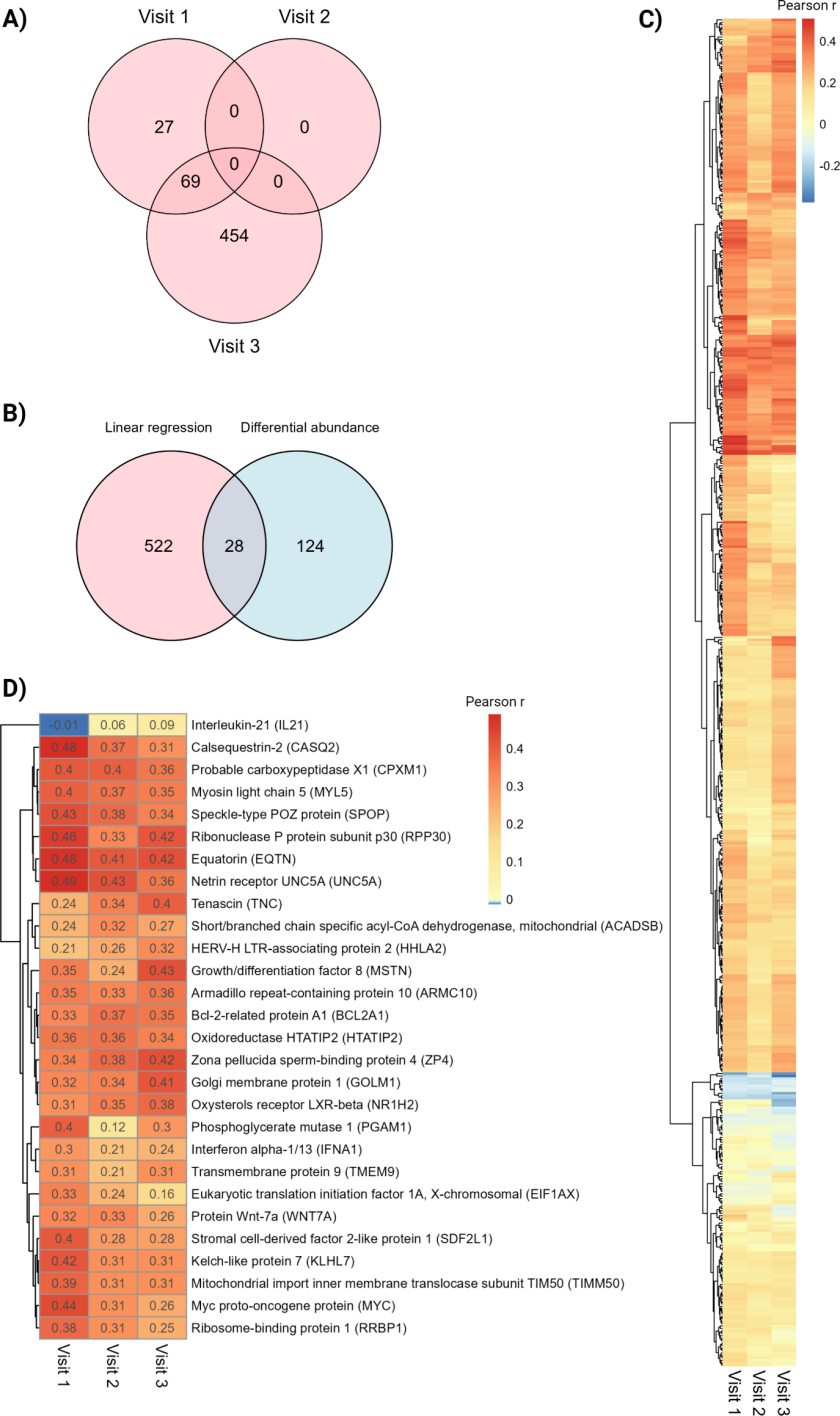
A) Venn diagram displaying the number of proteins significantly correlated with birth weight (z-score) at visits 1, 2, and 3. Ninty-six proteins were significantly associated with birth weight z-score at visit 1, 0 proteins at visit 2 and 523 proteins at visit 3. B) Twenty-eight proteins were correlated with birth weight and differentially abundant between LGA and AGA samples. C) Spearman’s Rank correlation coefficient between birth weight z-score and protein abundance at visits 1, 2, and 3 of the 28 proteins significant in both differential abundance analysis and linear regression analysis. D) Spearman’s Rank correlation coefficient (ρ) between birth weight z-score and protein abundance of the 550 significant proteins at either visit 1, 2, or 3.

The promising predictive ability to identify risk of LGA fetus already early in pregnancy might suggest that the infant’s birth weight is associated with the mother’s proteomic profile prior to conception or in early stages of pregnancy. Thus, one may hypothesize that the early maternal proteome primarily is a reflection of maternal general metabolic health,^61^ which again is linked to fetal growth. On the other hand, but not supported by our data, it could be anticipated that an adverse maternal metabolic profile and the corresponding plasma proteome would be further exacerbated by pregnancy itself, linking the maternal proteome during last trimester closer to fetal growth. There is a need to further investigate this association between the maternal plasma proteome in early versus late pregnancy and its associations with fetal growth.

### Examples of Proteins Associated with Birth Weight

Myosin light chain 5 (MYL5) was significantly elevated in women carrying LGA infants compared to women carrying AGA and SGA infants at all three visits (Figure 8A), and was also positively correlated with birth weight at all three visits in crude analyses, though not significant at visits 1 and 2 after adjusting for multiple testing (Figure 8B). Plotting protein abundance across gestation shows that the levels of MYL5 were generally stable throughout gestation (Figure 8C).

**Figure 8:**
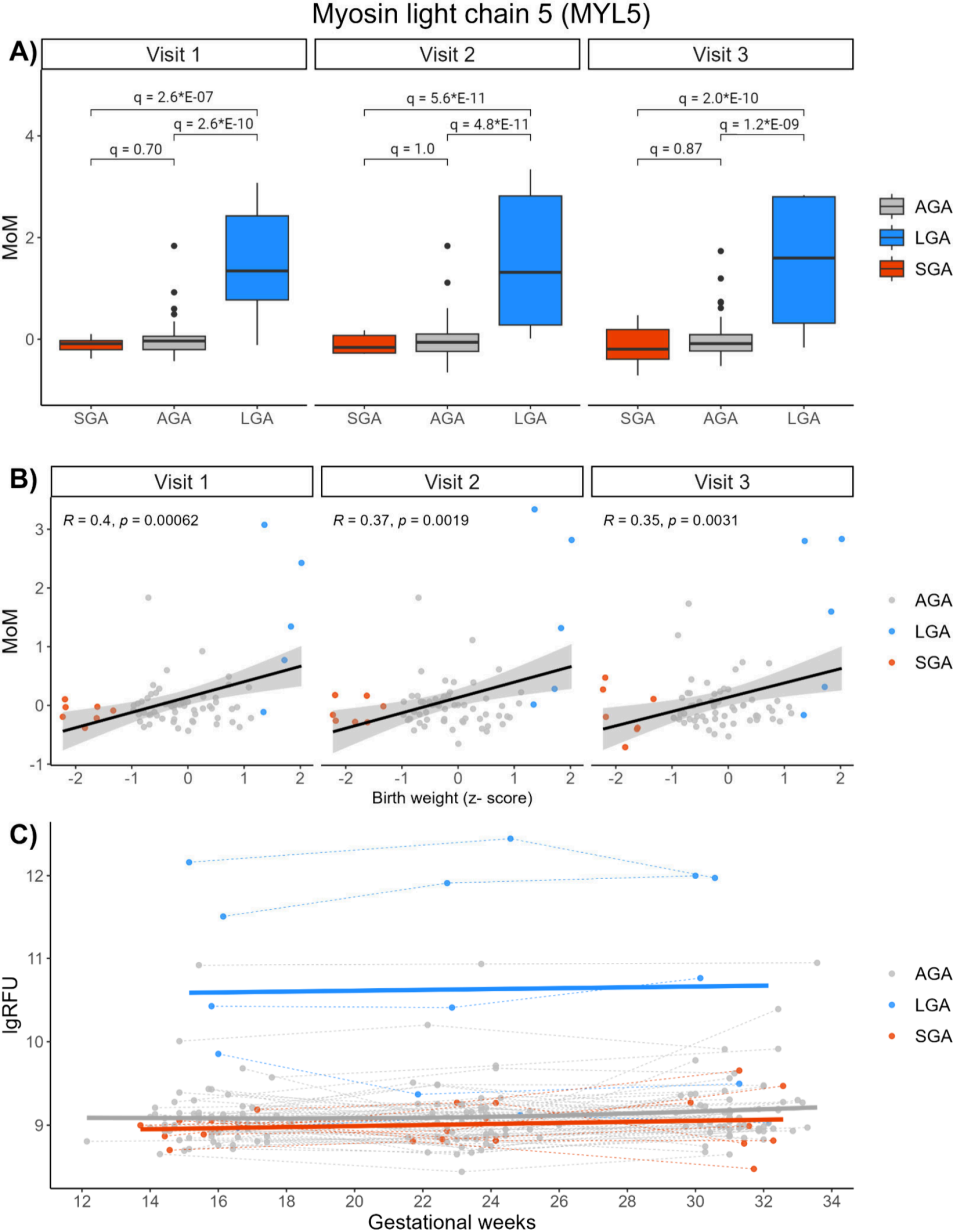
Protein expression patterns of myosin light chain 5 (MYL5). A) Box plots displaying MoM values of MYL5 at visits 1, 2, and 3, with q-values from moderated *t* tests between SGA, AGA, and LGA groups. B) Pearson correlation between MYL5MoM values and birth weight z-scores. C) Log RFU values of MYL5 across gestation. Mean curves within each birth weight group are fitted a generalized additive models.

By sequence similarity, MYL5 is reckoned to belong to the group of Regulatory Light Chains (RLC), which regulate the enzymatic activity of other myosin proteins.^62–64^ In association with Myosin-x (MYO10), MYL5 plays important roles during the cell cycle, and is crucial during mitotic spindle assembly and chromosome congression.^65^ Depleted levels of MYL5 have been linked to chromosome segregation errors^65^ and associated with several cancers,^66,67^ while overexpression of MYL5 has been associated with metastases of cervical cancer.^68^ Although MYL5 is the protein with the smallest q-value from the univariate tests (Figure 3), the trend was mainly driven by three of the five LGA participants. The variance of MYL5 abundance among the LGA samples was larger compared to most other significantly differential proteins (). A larger sample size of LGA participants is therefore needed to gain clarity of the MYL5 findings in the current analysis.

Similar patterns as for the MYL5 (higher abundance in LGA compared to AGA and SGA driven by two to three LGA samples) was observed for several other proteins with low q-values, such as NFKBIA, TIGIT, ARMC10, MTMR6, U2FA2, CRLF-1/CLC complex (CRLF1 CLCF1), and MSTN (). TGIT was also among the proteins with the largest variance among the LGA samples (). A higher number of LGA samples would therefore be needed to confirm that their increased abundance is associated with a large birth weight. Another interesting protein was Erythropoietin (EPO), which despite smaller fold change between groups had upregulated levels in all LGA samples and little within-group variance (). EPO was significantly elevated in women carrying LGA infants compared to women carrying AGA and SGA infants at visits 2 and 3 in crude analyses but was only significant between LGA and AGA at visit 3 after adjusting for multiple testing (Figure 9A). Levels of EPO were also positively correlated with the birth weight z-score in crude analysis at visit 3, but not after adjusting for multiple testing (Figure 9B). EPO is a glycoprotein hormone mainly involved in the production of red blood cells, but also involved in wound healing processes, vasoconstriction-dependent hypertension, angiogenesis and proliferation of smooth muscle fibers.^69–71^ Due to increased blood volume, the production of EPO increases 2–4 folds during pregnancy and reaches a plateau after 20 weeks of gestation.^71–75^ Levels of EPO have also been found to be higher in obese pregnant women than in normal weight women.^76^ In the current study, we found that EPO levels increased significantly throughout pregnancy with a steeper increase among the LGA participants (Figure 9C). Furthermore, EPO levels continued to increase after 20 weeks of gestation, and the difference between LGA and the SGA and AGA participants increased with gestational age. In humans, EPO does not cross the placenta,^77,78^ and the direct effect of EPO on fetal growth can therefore be questioned. However, as EPO is expressed by the placenta,^72,79^ increased levels in maternal blood could reflect increased placental size or increased placental metabolic activity. It is important to consider that while EPO has potential benefits for fetal growth through its actions on oxygen delivery and fetal erythropoiesis, the relationship is complex. For instance, high maternal EPO levels might suggest an underlying problem related to hypoxia,^80^ which in itself can be detrimental to fetal growth. Hence, maternal EPO levels could potentially serve as indicators of fetal growth. In clinical practice, abnormal EPO levels may prompt further investigation into the causes and provision of appropriate interventions to support both maternal health and fetal growth.

**Figure 9:**
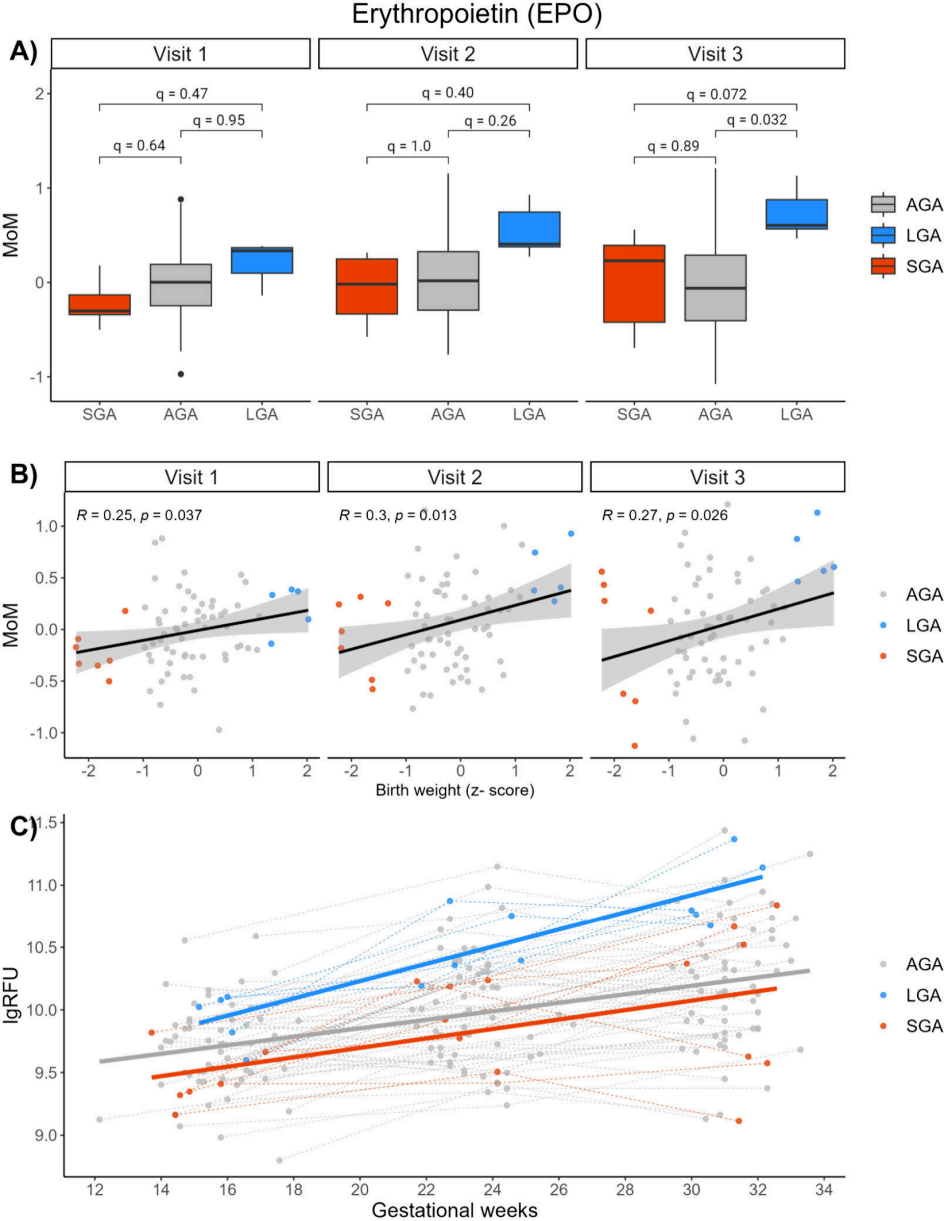
Protein expression patterns of erythropoietin (EPO). A) Box plots displaying MoM values of EPO at visits 1, 2, and 3, with q-values from moderated *t* tests between SGA, AGA and LGA groups. B) Pearson correlation between EPO MoM values and birth weight z-scores. C) Log RFU values (*y*-axis) of EPO across gestation (*x*-axis). Mean curves within each birth weight groups are fitted a generalized additive models.

### Biochemical Markers vs Ultrasound Biometry

Contrary to several previous studies that present predictive models including a few preselected biochemical markers combined with characteristics and fetal biometry,^16–22^ the models presented in the current analysis contain exclusively protein markers unbiasedly selected among nearly 5000 quantified proteins. The presented models are, to our knowledge, the currently best performing protein models for predicting LGA early in pregnancy and SGA in late pregnancy. The feasibility of a blood sample for protein quantification represents a considerable strength of models containing biochemical markers only. Nevertheless, with AUCs < 0.7 and relatively large 95% confidence intervals, the models presented in this study had lower predictive performance compared to previously published ultrasound based models. For example, Triunfo et al. (2017) predicted SGA (birth weight between the third and 10th centiles) based on ultrasound examinations with an AUC at 0.85 (95% confidence interval 0.82–0.89).^81^ However, the ultrasound scan was performed after 37 weeks gestation, considerably later compared to the samples in the current study. Ciobanu et al. (2019) predicted SGA (<10th percentile) at weeks 31–34 and 35–37 with ultrasound estimates, resulting in AUCs of 0.82 (95% confidence interval 0.81–0.83) and 0.88 (95% confidence interval 0.88–0.89) respectively, illustrating increasing predictive accuracy with gestational age.^82^

Although debated, several countries offer routine third trimester ultrasound scan as screening for fetal growth deviations.^83^ Such scans have high risk of false positives,^84,85^ and given that a common intervention in fetal growth restriction (FGR) pregnancies is delivery, preterm delivery of false positive SGA/FGR cases is highly disadvantageous. Induction of labor or cesarean delivery of false positive LGA cases is also unwarranted. High performing biochemical models independent of biophysical markers are therefore profitable to ensure correct diagnosis. Evaluation with biochemical models in addition ultrasound examination might also help distinguish fetuses with severe growth deviations from healthy LGA and SGA cases,^30^ and aid in clinical decisions regarding interventions or operative delivery when in doubt.

### Strengths and Limitations

Despite the low number of LGA and SGA cases in the current analysis, this is to our knowledge the largest study of protein biomarkers for birth weight in terms of number of measured proteins, inclusion of both LGA and SGA participants, and its longitudinal design. As far as we know, no former studies have investigated multiprotein prediction models for birthweight although researchers have expressed the need to use “omics” technologies to search for novel biomarkers of fetal growth.^30^ The SomaScan assay has a high dynamic range, meaning that it can quantify both high and low abundant proteins.^38^ This high dynamic range, in combination with a high number of proteins, gives SomaScan an advantage in explorative studies compared to other proteomic platforms such as ELISA and Mass Spectrometry (MS), which are limited to a lower number of proteins (ELISA) and give uncertain quantification of low abundant proteins (ELISA and MS).

Nevertheless, the study has several limitations. These are secondary analyses of a study that was originally designed as a pre-eclampsia prediction study, thus explaining the small numbers of SGA and LGA samples. Power calculations performed on the control samples of a previously published data set^34^ indicated an expected low power due to small samples sizes (). Still, the power calculations did not exclude the possibility of finding some differentially abundant proteins. For example, with FDR controlled at 0.2, we can expect a statistical power of 40% for identifying differentially abundant proteins if approximately 10% of all proteins are truly differential between the groups. The low number of SGA and LGA, and hence imbalance between cases and controls in the prediction models, also contributed to models with misleadingly high accuracy due to the high number of true negatives but low number of true positives, reflected by the low F1-scores and AUC-PR. Thus, this study should be regarded as an initial exploratory study paving the way for future large scale proteomic studies aiming to identify protein biomarkers of fetal growth. We encourage future studies to include a higher number of samples with low and high birth weights.

## Conclusion

Utilizing a longitudinal pregnancy cohort and comprehensive proteomic profiling of nearly 5000 proteins, we investigated the plasma proteome of mothers giving birth to SGA, AGA and LGA infants at three visits across gestation. The proposed models predicted LGA with good accuracy, while models predicting SGA gave moderate to poor accuracy. We further identified several proteins with differential abundance between AGA and LGA samples and proteins with positive correlation with birth weight across gestation with the strongest associations before 19 weeks of gestation. The relatively modest correlations between maternal proteins and infant birth weight indicate that the maternal proteome may not be ideal for prediction of fetal growth within the normal range but that it might be used to predict low or birth weight. Thus, we encourage future studies to include a higher number of participants with birth weights in lower or higher extremes.

## Supplementary Material









## Data Availability

The data sets generated and analyzed during the current study are available from the corresponding authors on reasonable request to Marie Cecilie P. Roland (mpaasche@ous-hf.no) or Trond M. Michelsen (t.m.michelsen@medisin.uio.no).

## Notes

**Supplementary Figure S1.** Histograms showing the distribution of demographic and clinical factors. **Supplementary Figure S2**. Receiver operating characteristic (ROC) curves for prediction of large for getational age (LGA) infants using the random forest machine learning algorithm.**Supplementary Figure S3**. Receiver operating characteristic (ROC) curves for prediction of large for getational age (LGA) infants using the elastic net machine learning algorithm. **Supplementary Figure S4**. Receiver operating characteristic (ROC) curves for prediction of small for getational age (SGA) infants using the random forest machine learning algorithm. **Supplementary Figure S5**. Receiver operating characteristic (ROC) curves for prediction of small for getational age (SGA) infants using the elastic net machine learning algorithm.**Supplementary Figure S6.** Histograms of p-values from moderated *t* tests between LGA and AGA at visit 1, 2, and 3. **Supplementary Figure S7.** Histograms of p-values from moderated *t* tests between SGA and AGA at visit 1, 2, and 3. **Supplementary Figure S8.** Histograms of p-values from linear regression analysis between birth weight z-score and protein abundance at visit 1, 2, and 3. **Supplementary Figure S9.** Heat map displaying the variance of each of the differential abundant proteins. **Supplementary Figure S10.** Protein expression patterns of NF-kappa-B inhibitor alpha (NKFBIA). **Supplementary Figure S11.** Protein expression patterns of T-cell immunoreceptor with Ig and ITIM domains (TIGIT). **Supplementary Figure S12.** Protein expression patterns of Armadillo repeat-containing protein 10 (ARMC10). **Supplementary Figure S13.** Protein expression patterns of Myotubularin-related protein 6 (MTMR6). **Supplementary Figure S14.** Protein expression patterns of Splicing factor U2AF 65 kDa subunit (U2AF2). **Supplementary Figure S15.** Protein expression patterns of Cytokine receptor-like factor 1:Cardiotrophin-like cytokine factor 1 Complex (CLF-1/CLC complex). **Supplementary Figure S16.** Protein expression patterns of Myostatin (MSTN). **Supplementary Figure S17.** Power calculation with fixed sample size, false discovery rate (FDR) and proportion of differentially abundant proteins (π_0_) based on data from Tarca, Romero, Benshalom-Tirosh, Than, Gudicha, Done, Pacora, Chaiworapongsa, Panaitescu, Tirosh, Gomez-Lopez, Draghici, Hassan, and Erez.^34^ With FDR = 0.05 and a sample size of six per group we expect <20% power if we assume that 10% of the proteins are differential and <55% power if we assume that 50% of the proteins are differential. **Supplementary Figure S18.** Power calculation with fixed sample size, false discovery rate (FDR) and proportion of differentially abundant proteins (π_0_) based on data from Tarca, Romero, Benshalom-Tirosh, Than, Gudicha, Done, Pacora, Chaiworapongsa, Panaitescu, Tirosh, Gomez-Lopez, Draghici, Hassan, and Erez.^34^ With FDR = 0.2 and a sample size of six per group we expect 40% power if we assume that 10% of the proteins are differential and >80% power if we assume that 50% of the proteins are differential. (PDF)
**Supplementary Table S1**. Results from differential abundance tests between large for gestational age (LGA) and adequate for gestational age (AGA) samples. (XLSX)
**Supplementary Table S2**. Results from differential abundance tests between large for gestational age (SGA) and adequate for gestational age (AGA) samples. (XLSX)
**Supplementary Table S3**. Results from linear regression analysis between protein abundance and birthweight (z-score). (XLSX)

## Notes

Participants in the STORK cohort signed a written informed consent based on the protocol approved by the Regional Committee for Medical and Health Research Ethics, Southern Norway, Oslo, Norway with reference number S-2014/224-0119a.
