# Supplementary material for: Maternal Plasma Proteins Associated with Birth Weight: A Longitudinal, Large Scale Proteomic Study
Source: J Proteome Res. 2025 May 5;24(7):3247–60. doi: 10.1021/acs.jproteome.4c00940 (PMC12235715; doi:10.1021/acs.jproteome.4c00940)
Supplement: Supplementary file 1 [file pr4c00940_si_001.pdf]

Supporting information: Maternal plasma proteins associated with birth weight: a longitudinal, large scale proteomic study

Authorship

Ina Jungersen Andresen[1,*], Ane Cecilie Westerberg[1,2], Marie Cecilie Paasche Roland[1,3], Manuela Zucknick[4] and Trond Melbye Michelsen[1,5,*]

Affiliations

[1]Department of Obstetrics, Division of Obstetrics and Gynecology, Oslo University Hospital, 0372, Oslo, Norway

[2] School of Health Sciences, Kristiania University College, Oslo, 0107, Norway

[3]Department of Medical Biochemistry, Oslo University Hospital, 0450, Oslo, Norway

[4]Department of Biostatistics, Oslo Centre for Biostatistics and Epidemiology, University of Oslo, 0372, Oslo, Norway

[5]Institute of Clinical Medicine, Faculty of Medicine, University of Oslo, 0372, Oslo, Norway

[*]Corresponding authors

Table of context

Supplementary Figure S1. Histograms showing the distribution of demographic and clinical factors.

Supplementary Figure S2. Receiver operating characteristic (ROC) curves for prediction of large for getational age (LGA) infants using the random forest machine learning algorithm.

Supplementary Figure S3. Receiver operating characteristic (ROC) curves for prediction of large for getational age (LGA) infants using the elastic net machine learning algorithm.

Supplementary Figure S4. Receiver operating characteristic (ROC) curves for prediction of small for getational age (SGA) infants using the random forest machine learning algorithm.

Supplementary Figure S5. Receiver operating characteristic (ROC) curves for prediction of small for getational age (SGA) infants using the elastic net machine learning algorithm.

Supplementary Figure S6. Histograms of p-values from moderated t-tests between LGA and AGA at visit 1, 2 and 3.

Supplementary Figure S7. Histograms of p-values from moderated t-tests between SGA and AGA at visit 1, 2 and 3.

Supplementary Figure S8. Histograms of p-values from linear regression analysis between birth weight z-score and protein abundance at visit 1, 2 and 3.

Supplementary Figure S9. Heat map displaying the variance of each of the differential abundant proteins.

Supplementary Figure S10. Protein expression patterns of NF-kappa-B inhibitor alpha (NKFBIA).

Supplementary Figure S11. Protein expression patterns of T-cell immunoreceptor with Ig and ITIM domains (TIGIT).

Supplementary Figure S12. Protein expression patterns of Armadillo repeat-containing protein 10 (ARMC10).

Supplementary Figure S13. Protein expression patterns of Myotubularin-related protein 6 (MTMR6).

Supplementary Figure S14. Protein expression patterns of Splicing factor U2AF 65 kDa subunit (U2AF2).

Supplementary Figure S15. Protein expression patterns of Cytokine receptor-like factor 1:Cardiotrophin-like cytokine factor 1 Complex (CLF-1/CLC complex).

Supplementary Figure S16. Protein expression patterns of Myostatin (MSTN).

Supplementary Figure S17. Power calculation with fixed sample size, false discovery rate (FDR) and proportion of differentially abundant proteins (π_0) based on data from Tarca, Romero, Benshalom-Tirosh, Than, Gudicha, Done, Pacora, Chaiworapongsa, Panaitescu, Tirosh, Gomez-Lopez, Draghici, Hassan and Erez [76]. With FDR = 0.05 and a sample size of six per group we expect <20% power if we

assume that 10% of the proteins are differential and <55% power if we assume that 50% of the proteins are differential.

Supplementary Figure S18. Power calculation with fixed sample size, false discovery rate (FDR) and proportion of differentially abundant proteins (π_0) based on data from Tarca, Romero, Benshalom-Tirosh, Than, Gudicha, Done, Pacora, Chaiworapongsa, Panaitescu, Tirosh, Gomez-Lopez, Draghici, Hassan and Erez [76]. With FDR = 0.2 and a sample size of six per group we expect 40% power if we assume that 10% of the proteins are differential and >80% power if we assume that 50% of the proteins are differential.

Supplementary Table S1. Results from differential abundance tests between large for gestational age (LGA) and adequate for gestational age (AGA) samples.

Supplementary Table S2. Results from differential abundance tests between large for gestational age (SGA) and adequate for gestational age (AGA) samples.

Supplementary Table S3. Results from linear regression analysis between protein abundance and birthweight (z-score).

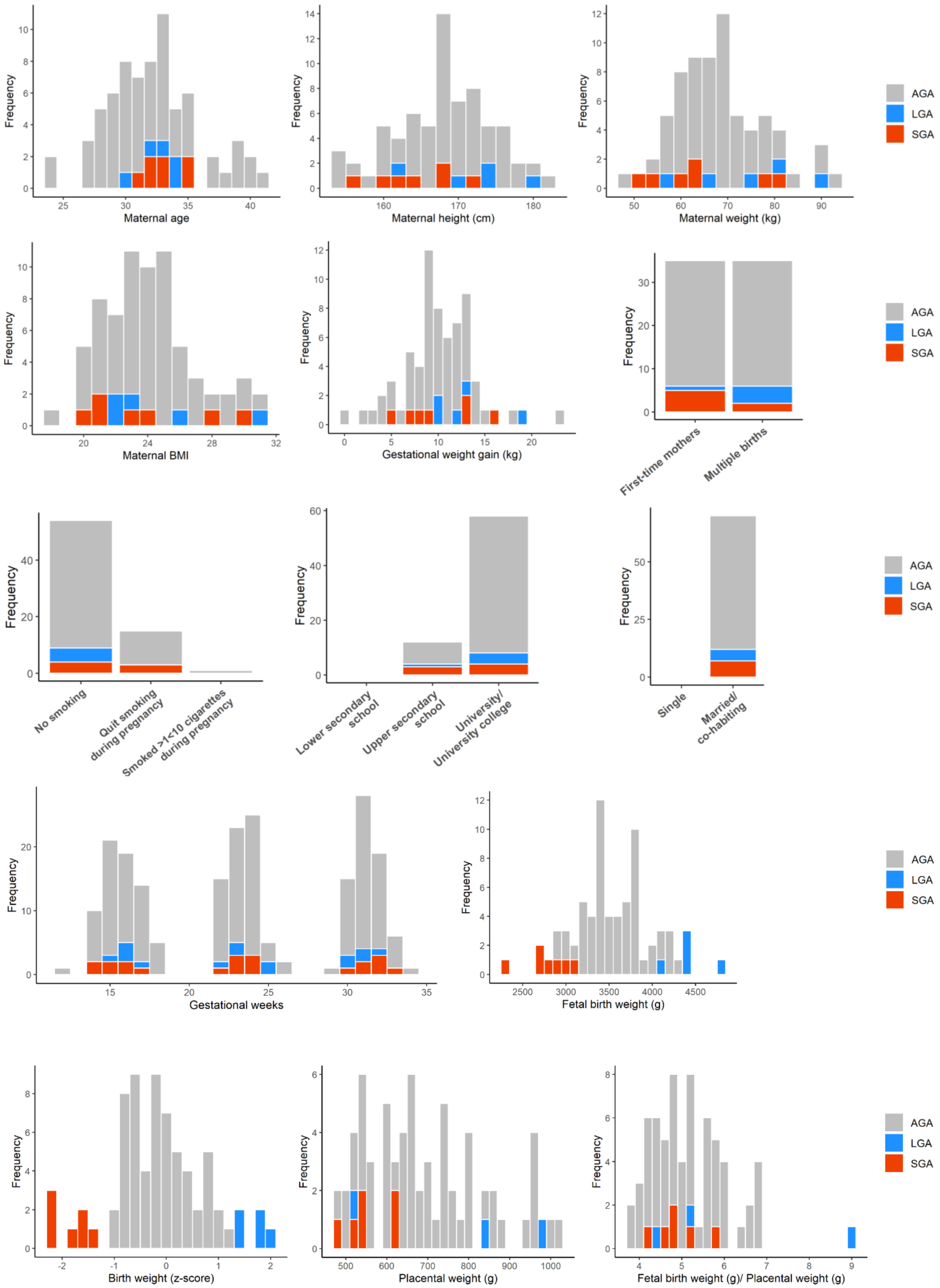
Frequency
Maternal age
Maternal height (cm)
Maternal weight (kg)
Maternal BMI
Gestational weight gain (kg)
First-time mothers
Multiple births
No smoking
Quit smoking during pregnancy
Smoked >1<10 cigarettes during pregnancy
Lower secondary school
Upper secondary school
University/ University college
Single
Married/ co-habiting
Gestational weeks
Fetal birth weight (g)
Birth weight (z-score)
Placental weight (g)
Fetal birth weight (g)/ Placental weight (g)
AGA
LGA
SGA

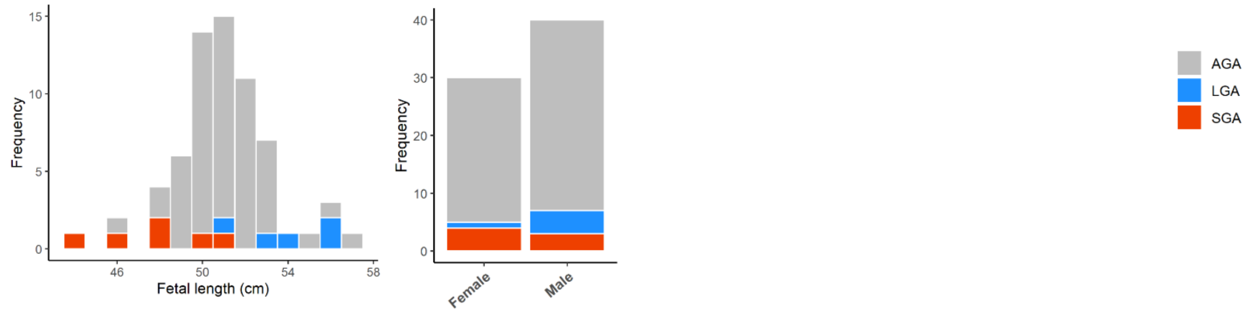

Supplementary Figure S1. *Distribution of demography and clinical factors.*

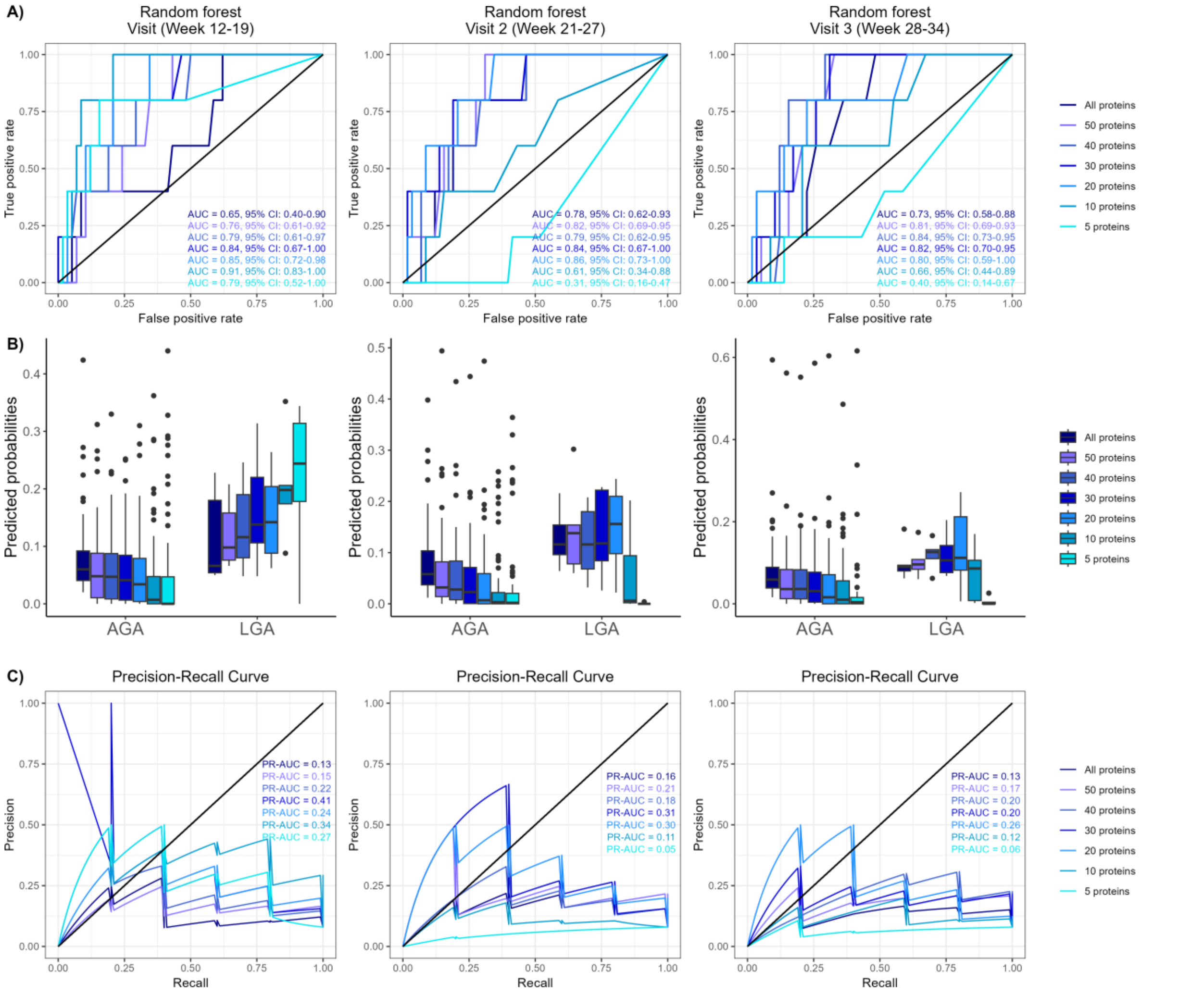

Supplementary Figure S2*. *A*)** *Receiver operating characteristic (ROC) curves,* ***B) *distribution of predicted probabilities, and* ***C***) *precision-Recall curves for prediction of large for getational age (LGA) infants using the random forest machine learning algorithm. Models were trained with leave-one-out cross-validation and all 4565 proteins passing quality check, and 50 to 5 proteins selected by moderated t-tests between LGA and adequate for gestational age (AGA) at each cross validation iteration.*

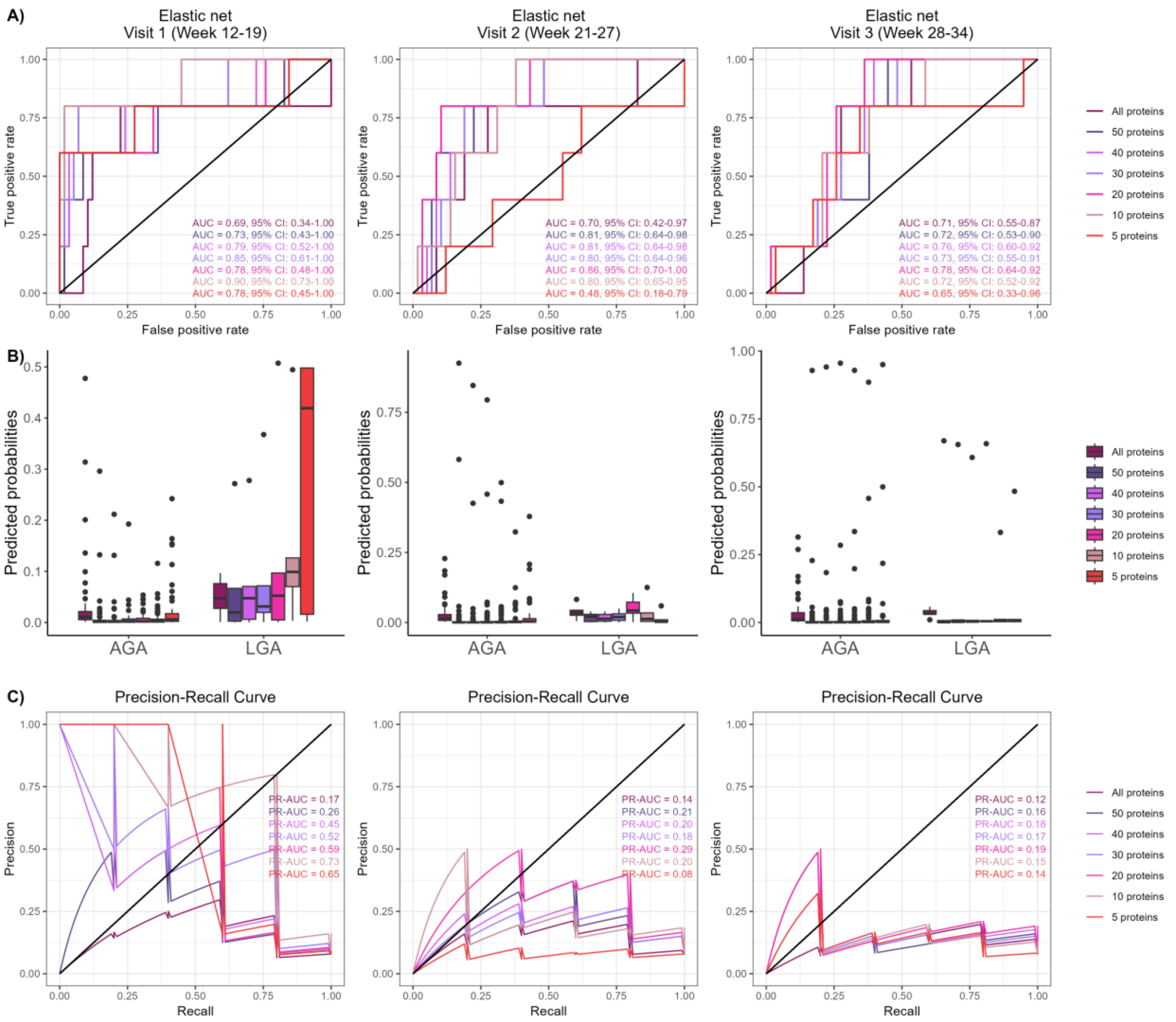

Supplementary Figure S3. ***A****) Receiver operating characteristic (ROC) curves,* ***B****) distribution of predicted probabilities, and* ***C****) precision-Recall curves for prediction of large for getational age (LGA) infants using the elastic net machine learning algorithm. Models were trained with leave-one-out cross-validation and all 4565 proteins passing quality check, and 50 to 5 proteins selected by moderated t-tests between LGA and adequate for gestational age (AGA) at each cross validation iteration.*

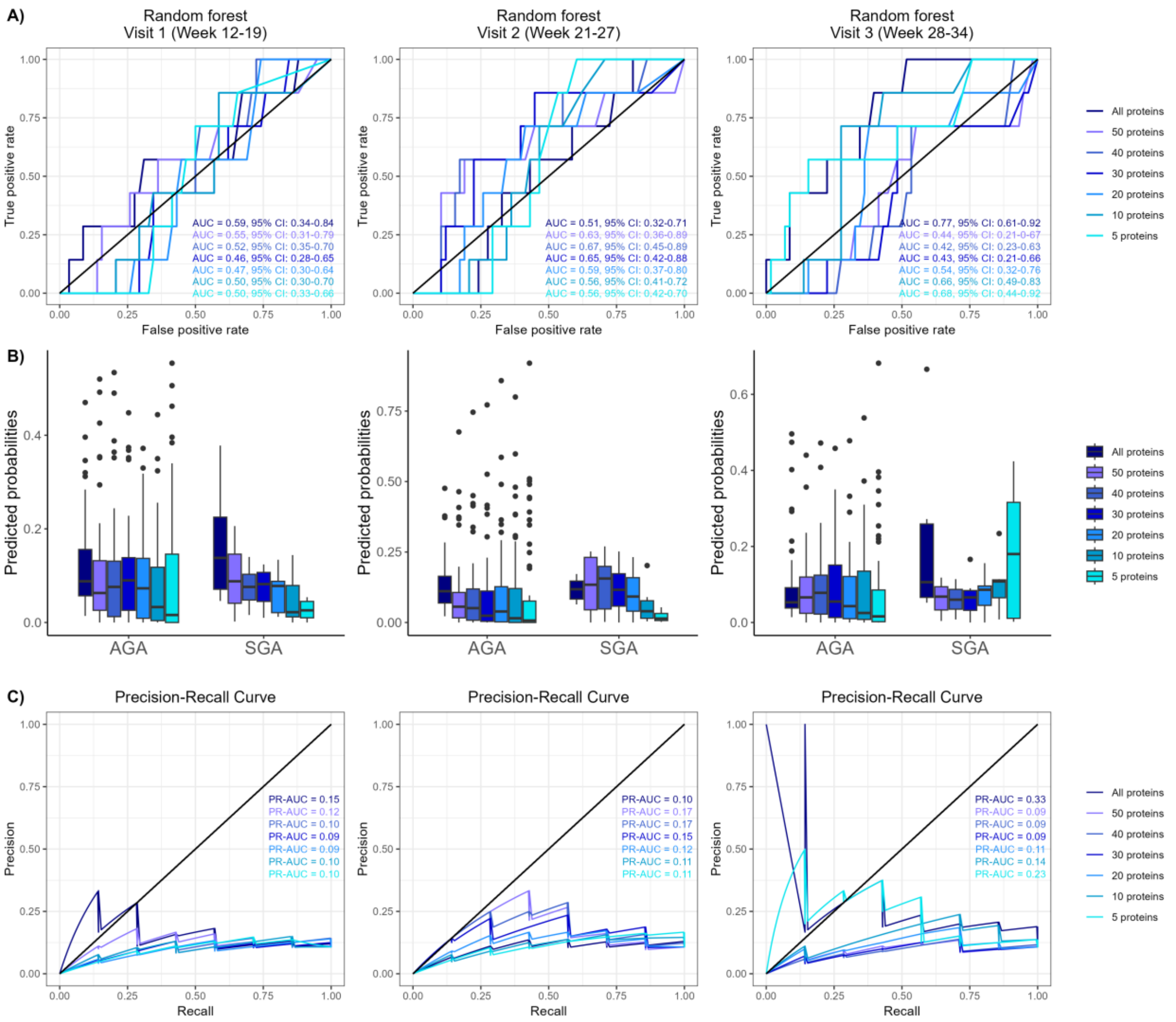

Supplementary Figure S4*. *A*)** *Receiver operating characteristic (ROC) curves,* ***B*) distribution of predicted probabilities, and* ***C****) precision-Recall curves for prediction of small for getational age (SGA) infants using the random forest machine learning algorithm. Models were trained with leave-one-out cross-validation and all 4565 proteins passing quality check, and 50 to 5 proteins selected by moderated t-tests between SGA and adequate for gestational age (AGA) at each cross validation iteration.*

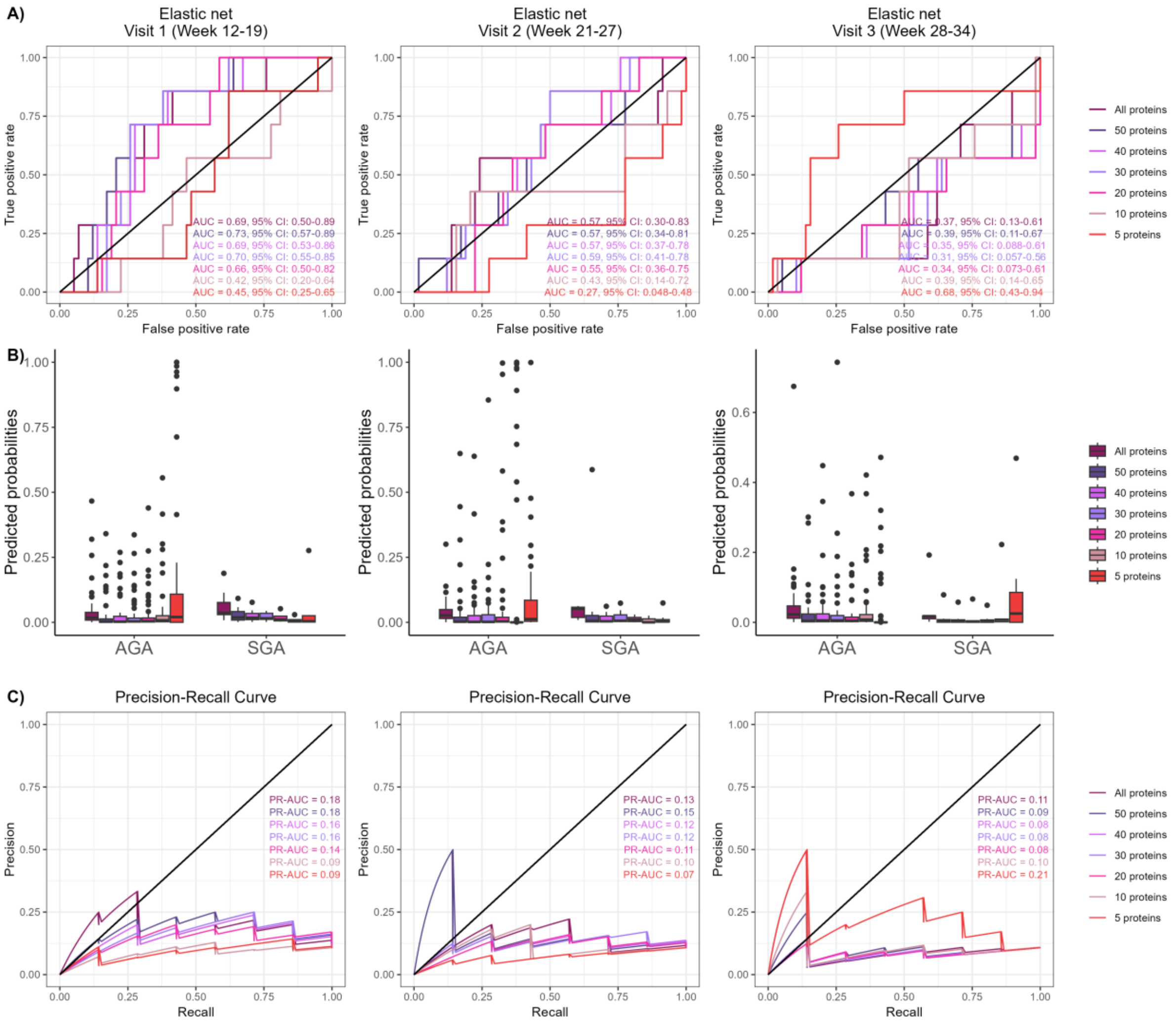

***Supplementary Figure S5*. *A*)** *Receiver operating characteristic (ROC) curves,* ***B*)** *distribution of predicted probabilities, and* ***C*)** *precision-Recall curves for prediction of small for getational age (SGA) infants using the elastic net machine learning algorithm. Models were trained with leave-one-out cross-validation and all 4565 proteins passing quality check, and 50 to 5 proteins selected by moderated t-tests between SGA and adequate for gestational age (AGA) at each cross validation iteration.*

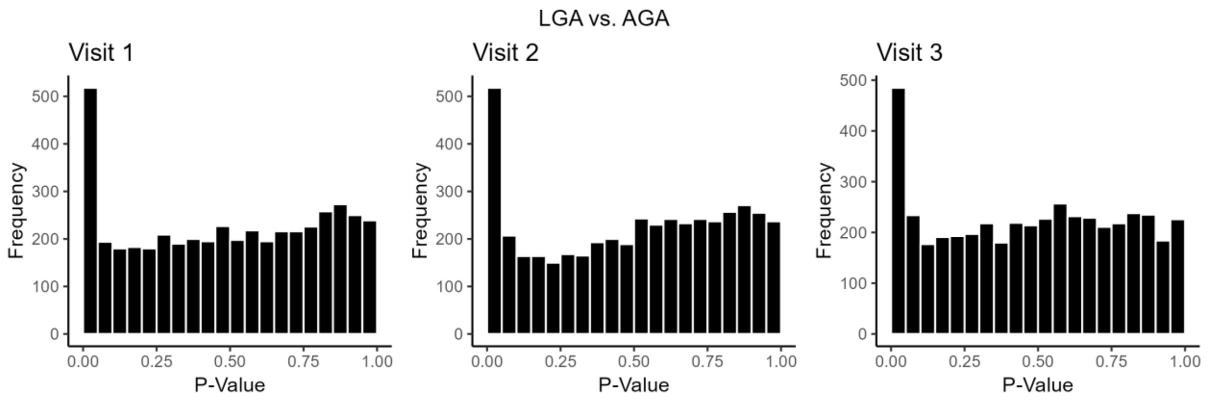

Supplementary *Figure S6.* *Histograms of p-values from moderated t-tests between LGA and AGA at visit 1, 2 and 3.*

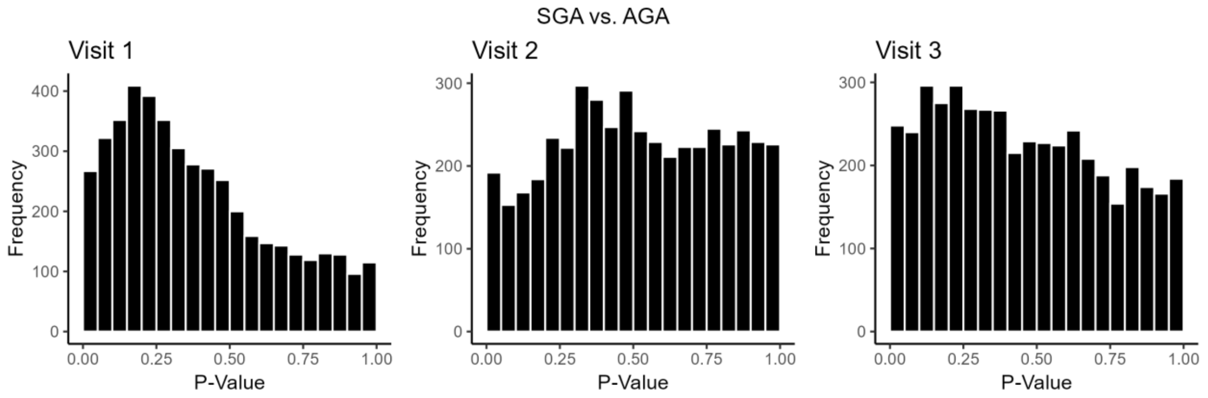

Supplementary *Figure S7.* *Histograms of p-values from moderated t-tests between SGA and AGA at visit 1, 2 and 3.*

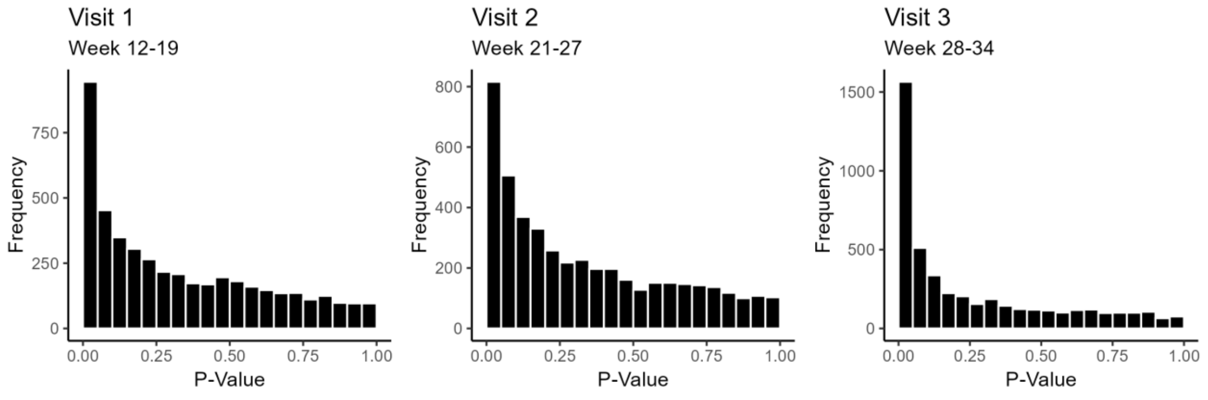

Supplementary *Figure S8.* *Histograms of p-values from linear regression analysis between birth weight z-score and protein abundance at visit 1, 2 and 3.*

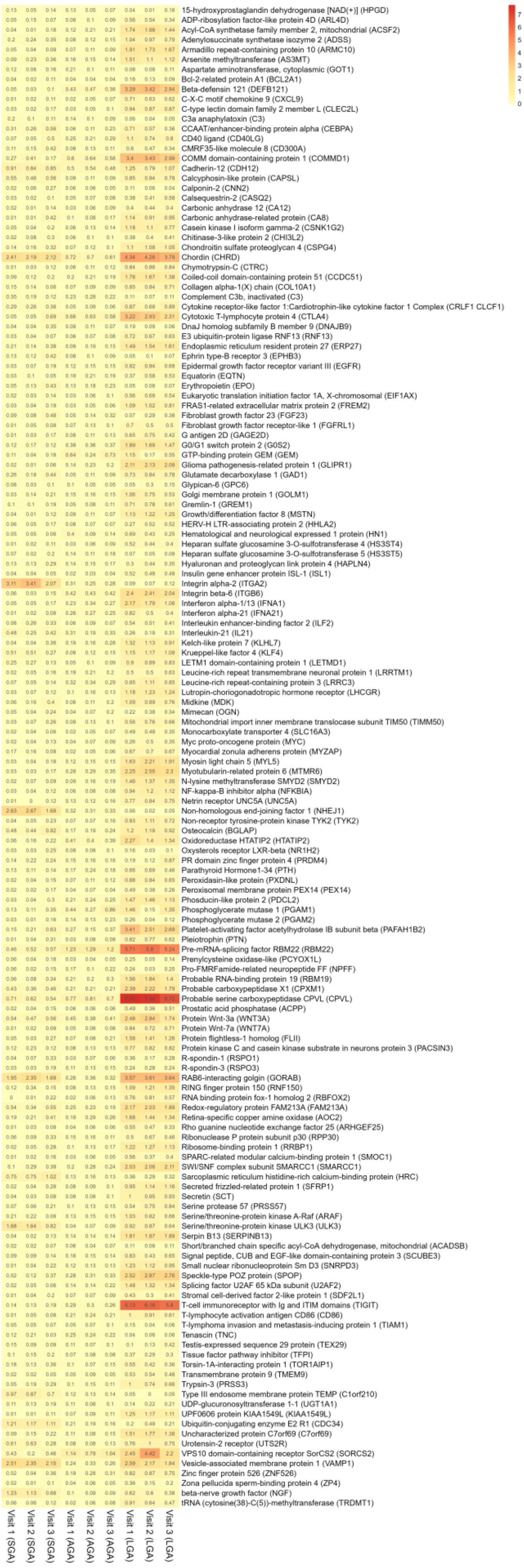
15-hydroxyprostaglandin dehydrogenase [NAD(+)] (HPGD)
ADP-ribosylation factor-like protein 4D (ARL4D)
Acyl-CoA synthetase family member 2, mitochondrial (ACSF2)
Adenylosuccinate synthetase isozyme 2 (ADSS)
Armadillo repeat-containing protein 10 (ARMC10)
Arsenite methyltransferase (AS3MT)
Aspartate aminotransferase, cytoplasmic (GOT1)
Bcl-2-related protein A1 (BCL2A1)
Beta-defensin 121 (DEFB121)
C-X-C motif chemokine 9 (CXCL9)
C-type lectin domain family 2 member L (CLEC2L)
C3a anaphylatoxin (C3)
CCAAT/enhancer-binding protein alpha (CEBPA)
CD40 ligand (CD40LG)
CMRF35-like molecule 8 (CD300A)
COMM domain-containing protein 1 (COMMD1)
Cadherin-12 (CDH12)
Calcyphosin-like protein (CAPSL)
Calponin-2 (CNN2)
Calsequestrin-2 (CASQ2)
Carbonic anhydrase 12 (CA12)
Carbonic anhydrase-related protein (CA8)
Casein kinase I isoform gamma-2 (CSNK1G2)
Chitinase-3-like protein 2 (CHI3L2)
Chondroitin sulfate proteoglycan 4 (CSPG4)
Chordin (CHRD)
Chymotrypsin-C (CTRC)
Coiled-coil domain-containing protein 51 (CCDC51)
Collagen alpha-1(X) chain (COL10A1)
Complement C3b, inactivated (C3)
Cytokine receptor-like factor 1:Cardiotrophin-like cytokine factor 1 Complex (CRLF1 CLCF1)
Cytotoxic T-lymphocyte protein 4 (CTLA4)
DnaJ homolog subfamily B member 9 (DNAJB9)
E3 ubiquitin-protein ligase RNF13 (RNF13)
Endoplasmic reticulum resident protein 27 (ERP27)
Ephrin type-B receptor 3 (EPHB3)
Epidermal growth factor receptor variant III (EGFR)
Equatorin (EQTN)
Erythropoietin (EPO)
Eukaryotic translation initiation factor 1A, X-chromosomal (EIF1AX)
FRAS1-related extracellular matrix protein 2 (FREM2)
Fibroblast growth factor 23 (FGF23)
Fibroblast growth factor receptor-like 1 (FGFRL1)
G antigen 2D (GAGE2D)
G0/G1 switch protein 2 (G0S2)
GTP-binding protein GEM (GEM)
Glioma pathogenesis-related protein 1 (GLIPR1)
Glutamate decarboxylase 1 (GAD1)
Glypican-6 (GPC6)
Golgi membrane protein 1 (GOLM1)
Gremlin-1 (GREM1)
Growth/differentiation factor 8 (MSTN)
HERV-H LTR-associating protein 2 (HHLA2)
Hematological and neurological expressed 1 protein (HN1)
Heparan sulfate glucosamine 3-O-sulfotransferase 4 (HS3ST4)
Heparan sulfate glucosamine 3-O-sulfotransferase 5 (HS3ST5)
Hyaluronan and proteoglycan link protein 4 (HAPLN4)
Insulin gene enhancer protein ISL-1 (ISL1)
Integrin alpha-2 (ITGA2)
Integrin beta-6 (ITGB6)
Interferon alpha-1/13 (IFNA1)
Interferon alpha-21 (IFNA21)
Interleukin enhancer-binding factor 2 (ILF2)
Interleukin-21 (IL21)
Kelch-like protein 7 (KLHL7)
Krueppel-like factor 4 (KLF4)
LETM1 domain-containing protein 1 (LETMD1)
Leucine-rich repeat transmembrane neuronal protein 1 (LRRTM1)
Leucine-rich repeat-containing protein 3 (LRRC3)
Lutropin-choriogonadotropic hormone receptor (LHCGR)
Midkine (MDK)
Mimecan (OGN)
Mitochondrial import inner membrane translocase subunit TIM50 (TIMM50)
Monocarboxylate transporter 4 (SLC16A3)
Myc proto-oncogene protein (MYC)
Myocardial zonula adherens protein (MYZAP)
Myosin light chain 5 (MYL5)
Myotubularin-related protein 6 (MTMR6)
N-lysine methyltransferase SMYD2 (SMYD2)
NF-kappa-B inhibitor alpha (NFKBIA)
Netrin receptor UNC5A (UNC5A)
Non-homologous end-joining factor 1 (NHEJ1)
Non-receptor tyrosine-protein kinase TYK2 (TYK2)
Osteocalcin (BGLAP)
Oxidoreductase HTATIP2 (HTATIP2)
Oxysterols receptor LXR-beta (NR1H2)
PR domain zinc finger protein 4 (PRDM4)
Parathyroid Hormone1-34 (PTH)
Peroxidasin-like protein (PXDNL)
Peroxisomal membrane protein PEX14 (PEX14)
Phosducin-like protein 2 (PDCL2)
Phosphoglycerate mutase 1 (PGAM1)
Phosphoglycerate mutase 2 (PGAM2)
Platelet-activating factor acetylhydrolase IB subunit beta (PAFAH1B2)
Pleiotrophin (PTN)
Pre-mRNA-splicing factor RBM22 (RBM22)
Prenylcysteine oxidase-like (PCYOX1L)
Pro-FMRFamide-related neuropeptide FF (NPFF)
Probable RNA-binding protein 19 (RBM19)
Probable carboxypeptidase X1 (CPXM1)
Probable serine carboxypeptidase CPVL (CPVL)
Prostatic acid phosphatase (ACPP)
Protein Wnt-3a (WNT3A)
Protein Wnt-7a (WNT7A)
Protein flightless-1 homolog (FLII)
Protein kinase C and casein kinase substrate in neurons protein 3 (PACSIN3)
R-spondin-1 (RSPO1)
R-spondin-3 (RSPO3)
RAB6-interacting golgin (GORAB)
RING finger protein 150 (RNF150)
RNA binding protein fox-1 homolog 2 (RBFOX2)
Redox-regulatory protein FAM213A (FAM213A)
Retina-specific copper amine oxidase (AOC2)
Rho guanine nucleotide exchange factor 25 (ARHGEF25)
Ribonuclease P protein subunit p30 (RPP30)
Ribosome-binding protein 1 (RRBP1)
SPARC-related modular calcium-binding protein 1 (SMOC1)
SWI/SNF complex subunit SMARCC1 (SMARCC1)
Sarcoplasmic reticulum histidine-rich calcium-binding protein (HRC)
Secreted frizzled-related protein 1 (SFRP1)
Secretin (SCT)
Serine protease 57 (PRSS57)
Serine/threonine-protein kinase A-Raf (ARAF)
Serine/threonine-protein kinase ULK3 (ULK3)
Serpin B13 (SERPINB13)
Short/branched chain specific acyl-CoA dehydrogenase, mitochondrial (ACADSB)
Signal peptide, CUB and EGF-like domain-containing protein 3 (SCUBE3)
Small nuclear ribonucleoprotein Sm D3 (SNRPD3)
Speckle-type POZ protein (SPOP)
Splicing factor U2AF 65 kDa subunit (U2AF2)
Stromal cell-derived factor 2-like protein 1 (SDF2L1)
T-cell immunoreceptor with Ig and ITIM domains (TIGIT)
T-lymphocyte activation antigen CD86 (CD86)
T-lymphoma invasion and metastasis-inducing protein 1 (TIAM1)
Tenascin (TNC)
Testis-expressed sequence 29 protein (TEX29)
Tissue factor pathway inhibitor (TFPI)
Torsin-1A-interacting protein 1 (TOR1AIP1)
Transmembrane protein 9 (TMEM9)
Trypsin-3 (PRSS3)
Type III endosome membrane protein TEMP (C1orf210)
UDP-glucuronosyltransferase 1-1 (UGT1A1)
UPF0606 protein KIAA1549L (KIAA1549L)
Ubiquitin-conjugating enzyme E2 R1 (CDC34)
Uncharacterized protein C7orf69 (C7orf69)
Urotensin-2 receptor (UTS2R)
VPS10 domain-containing receptor SorCS2 (SORCS2)
Vesicle-associated membrane protein 1 (VAMP1)
Zinc finger protein 526 (ZNF526)
Zona pellucida sperm-binding protein 4 (ZP4)
beta-nerve growth factor (NGF)
tRNA (cytosine(38)-C(5))-methyltransferase (TRDMT1)
Visit 1 (SGA)
Visit 2 (SGA)
Visit 3 (SGA)
Visit 1 (AGA)
Visit 2 (AGA)
Visit 3 (AGA)
Visit 1 (LGA)
Visit 2 (LGA)
Visit 3 (LGA)
7
6
5
4
3
2
1
0

Supplementary *Figure S9.* *Heat map displaying the variance of each of the 152 differential abundant proteins (148 proteins when testing LGA against AGA and four proteins when testing SGA against AGA), within each birth weight group at each visit. Proteins are given in alphabetical order.*

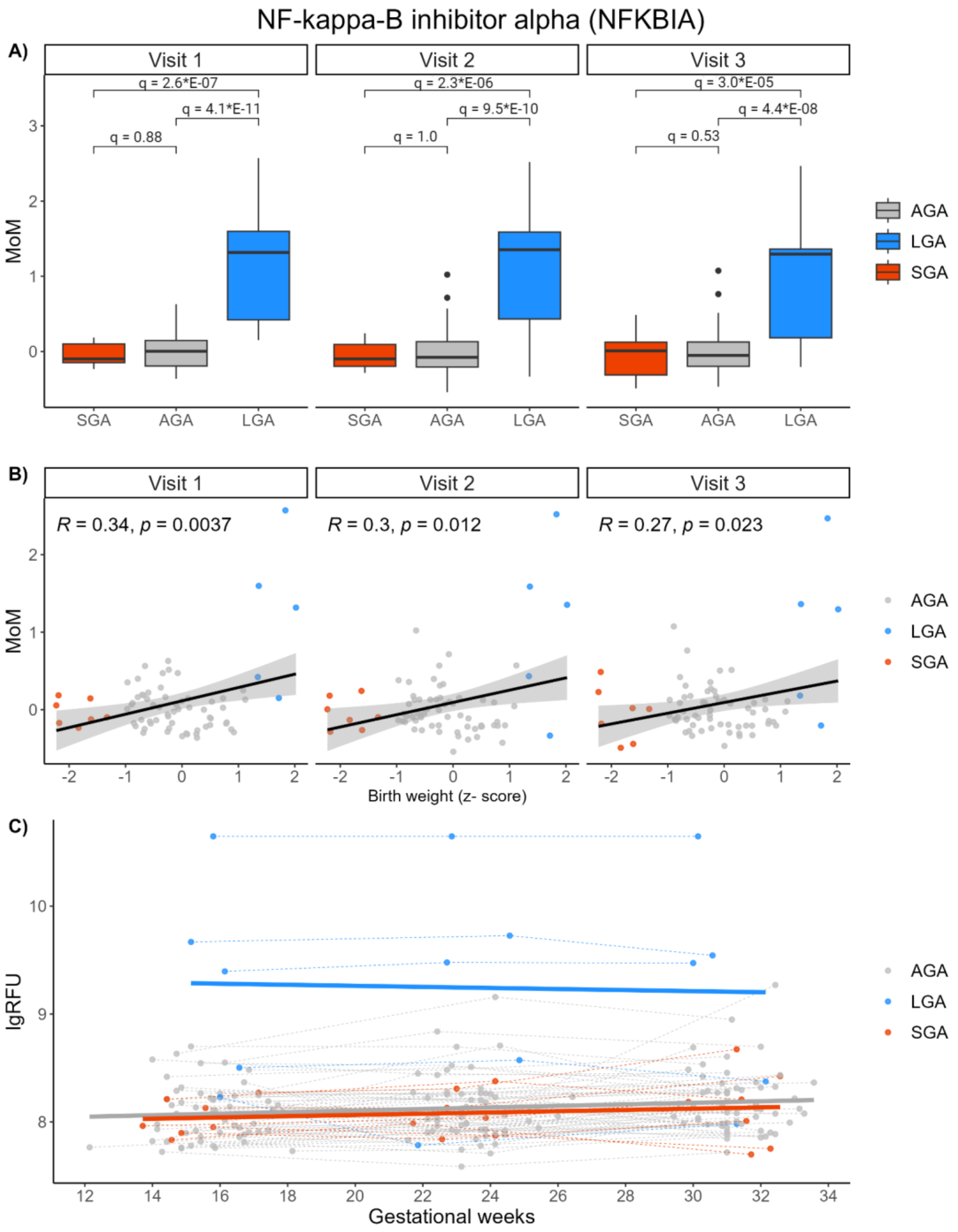

Supplementary *Figure S10.* *Protein expression patterns of NF-kappa-B inhibitor alpha (NKFBIA).* ***A****) Box plots displaying MoM values of NKFBIA at visit 1, 2 and 3, with q-values from moderated t-tests between SGA, AGA and LGA groups.* ***B****) Pearson correlation between NKFBIA MoM values and birth weight z-scores.* ***C****) Log RFU values of NKFBIA across gestation. Mean curves within each birth weight groups are fitted a generalized additive models.*

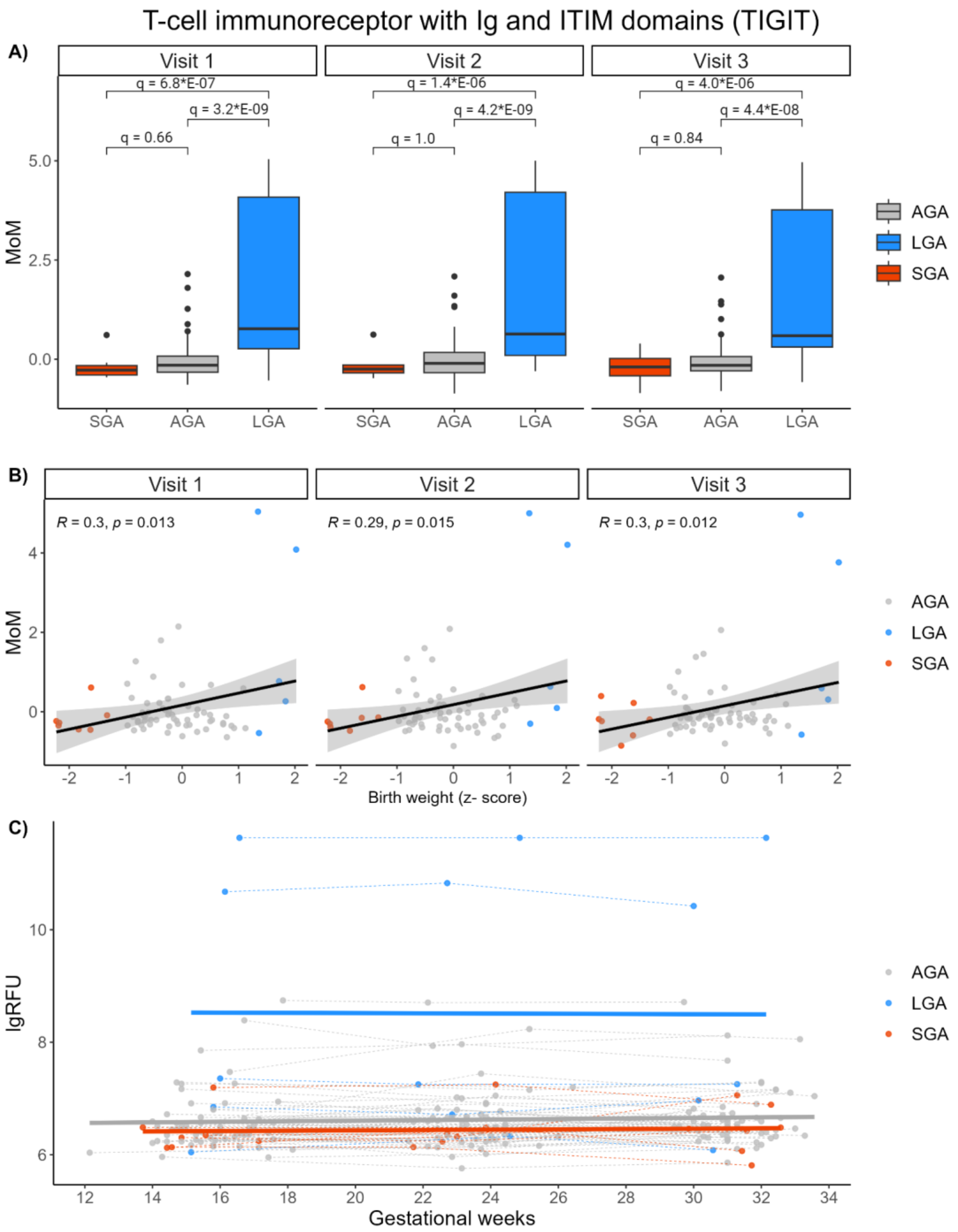

Supplementary *Figure S11.* *Protein expression patterns of T-cell immunoreceptor with Ig and ITIM domains (TIGIT).* ***A****) Box plots displaying MoM values of TIGIT at visit 1, 2 and 3, with q-values from moderated t-tests between SGA, AGA and LGA groups.* ***B****) Pearson correlation between TIGIT MoM values and birth weight z-scores.* ***C****) Log RFU values of TIGIT across gestation. Mean curves within each birth weight groups are fitted a generalized additive models.*

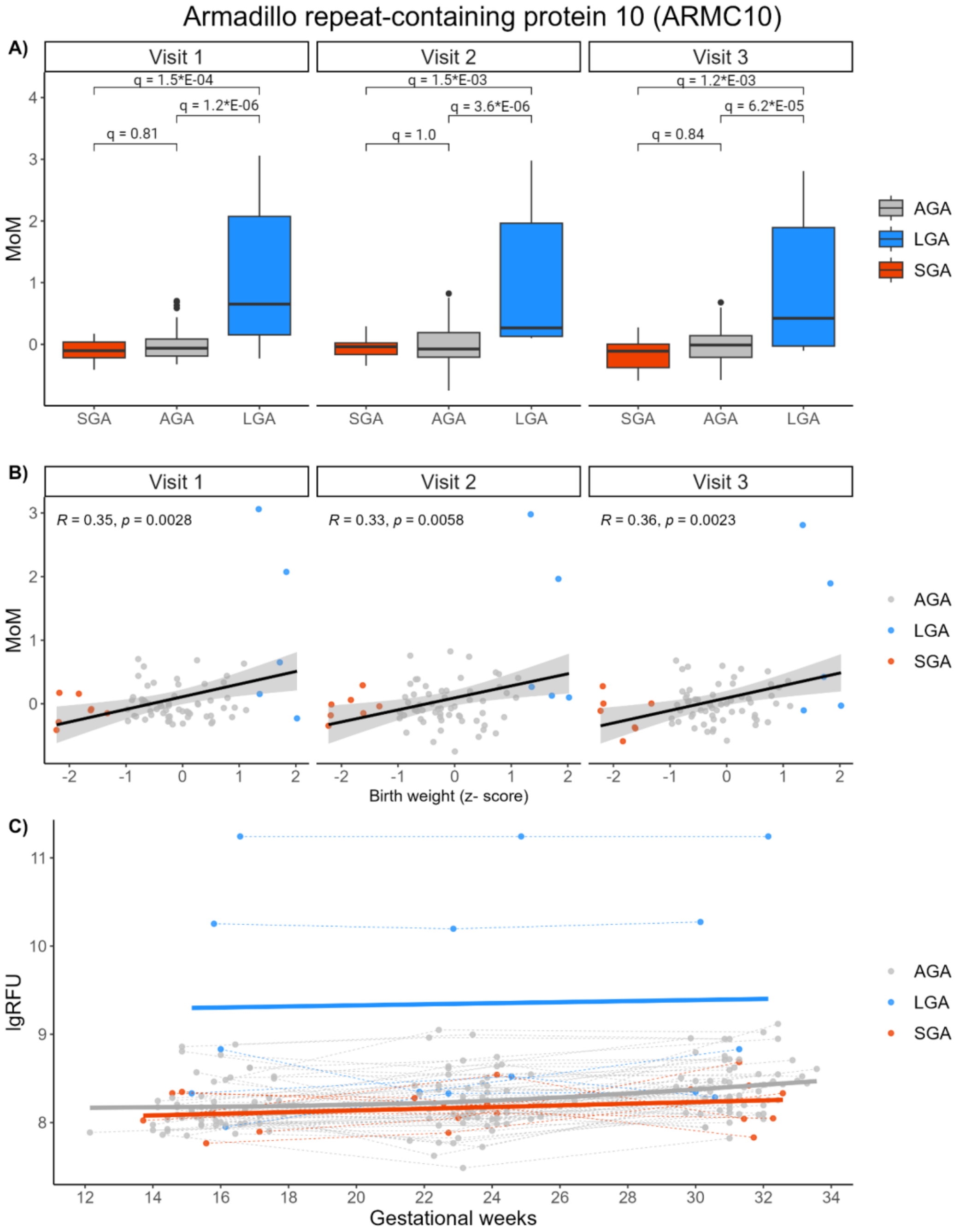

Supplementary *Figure S12.* *Protein expression patterns of Armadillo repeat-containing protein 10 (ARMC10). **A**) Box plots displaying MoM values of ARMC10 at visit 1, 2 and 3, with q-values from moderated t-tests between SGA, AGA and LGA groups. **B**) Pearson correlation between ARMC10 MoM values and birth weight z-scores. **C**) Log RFU values of ARMC10 across gestation. Mean curves within each birth weight groups are fitted a generalized additive models.*

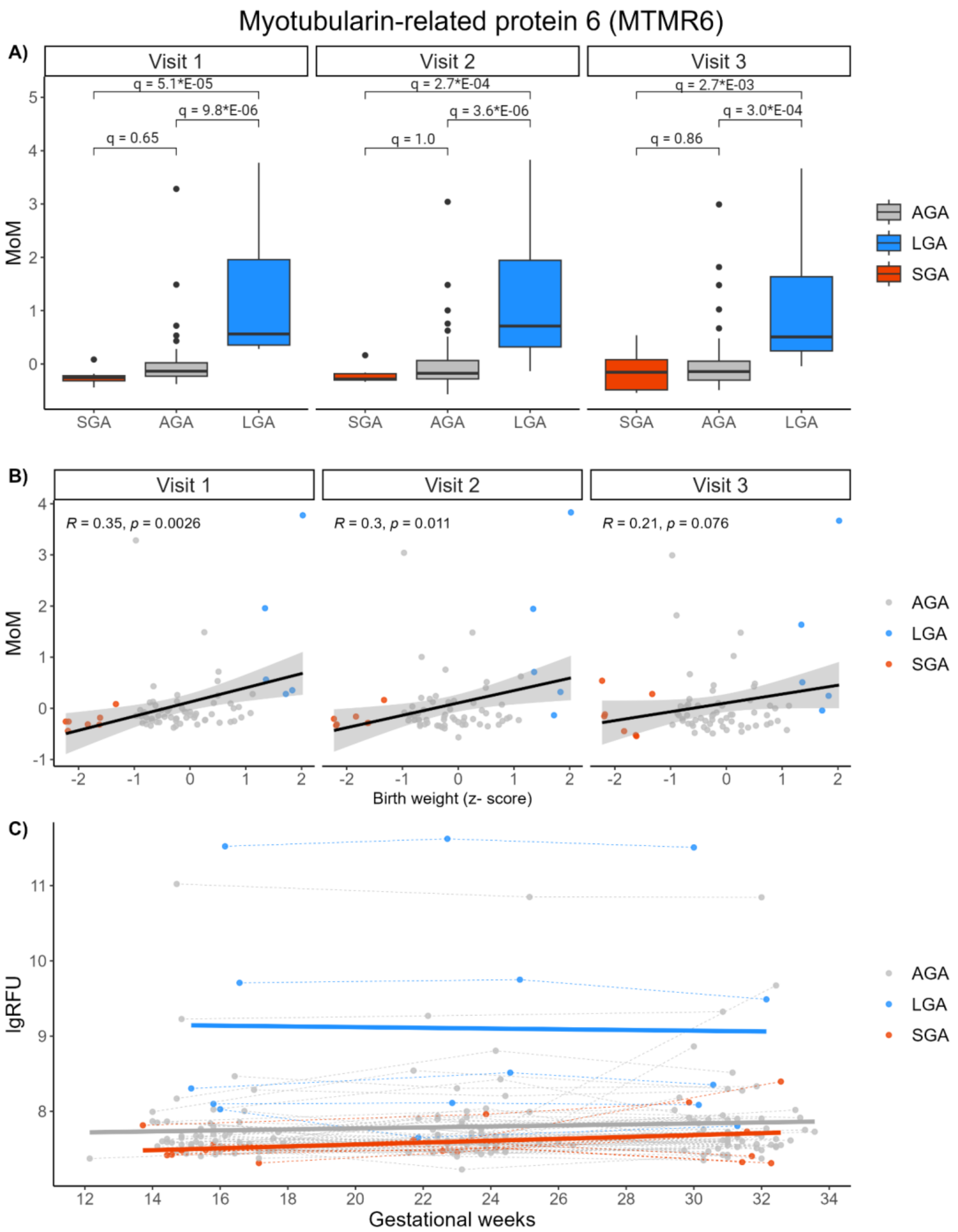

Supplementary *Figure S13.* *Protein expression patterns of Myotubularin-related protein 6 (MTMR6).* ***A****) Box plots displaying MoM values of MTMR6 at visit 1, 2 and 3, with q-values from moderated t-tests between SGA, AGA and LGA groups.* ***B****) Pearson correlation between MTMR6 MoM values and birth weight z-scores.* ***C****) Log RFU values of MTMR6 across gestation. Mean curves within each birth weight groups are fitted a generalized additive models.*

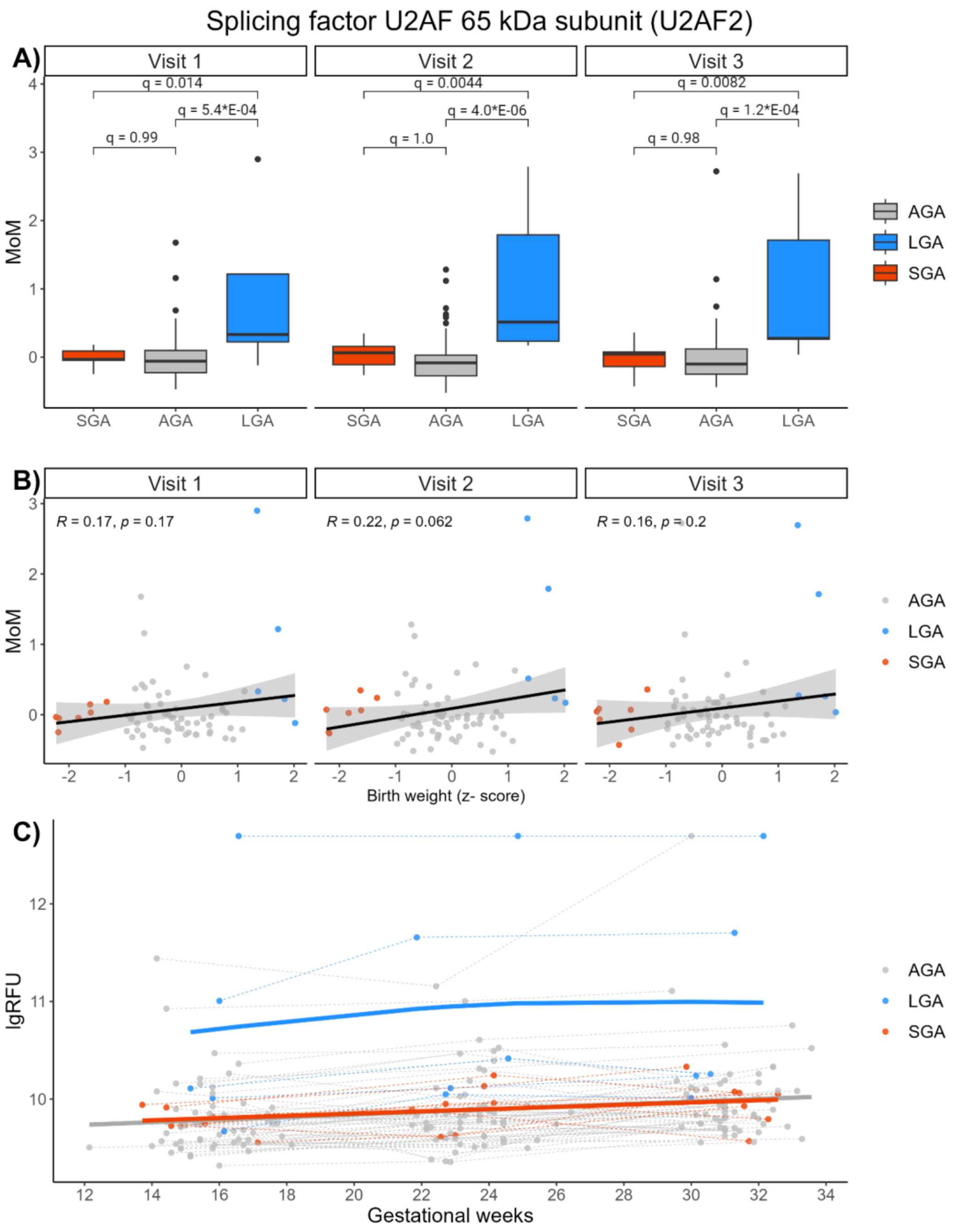

Supplementary ***Figure S14.*** *Protein expression patterns of Splicing factor U2AF 65 kDa subunit (U2AF2).* ***A****) Box plots displaying MoM values of U2AF2 at visit 1, 2 and 3, with q-values from moderated t-tests between SGA, AGA and LGA groups.* ***B****) Pearson correlation between U2AF2 MoM values and birth weight z-scores.* ***C****) Log RFU values of U2AF2 across gestation. Mean curves within each birth weight groups are fitted a generalized additive models.*

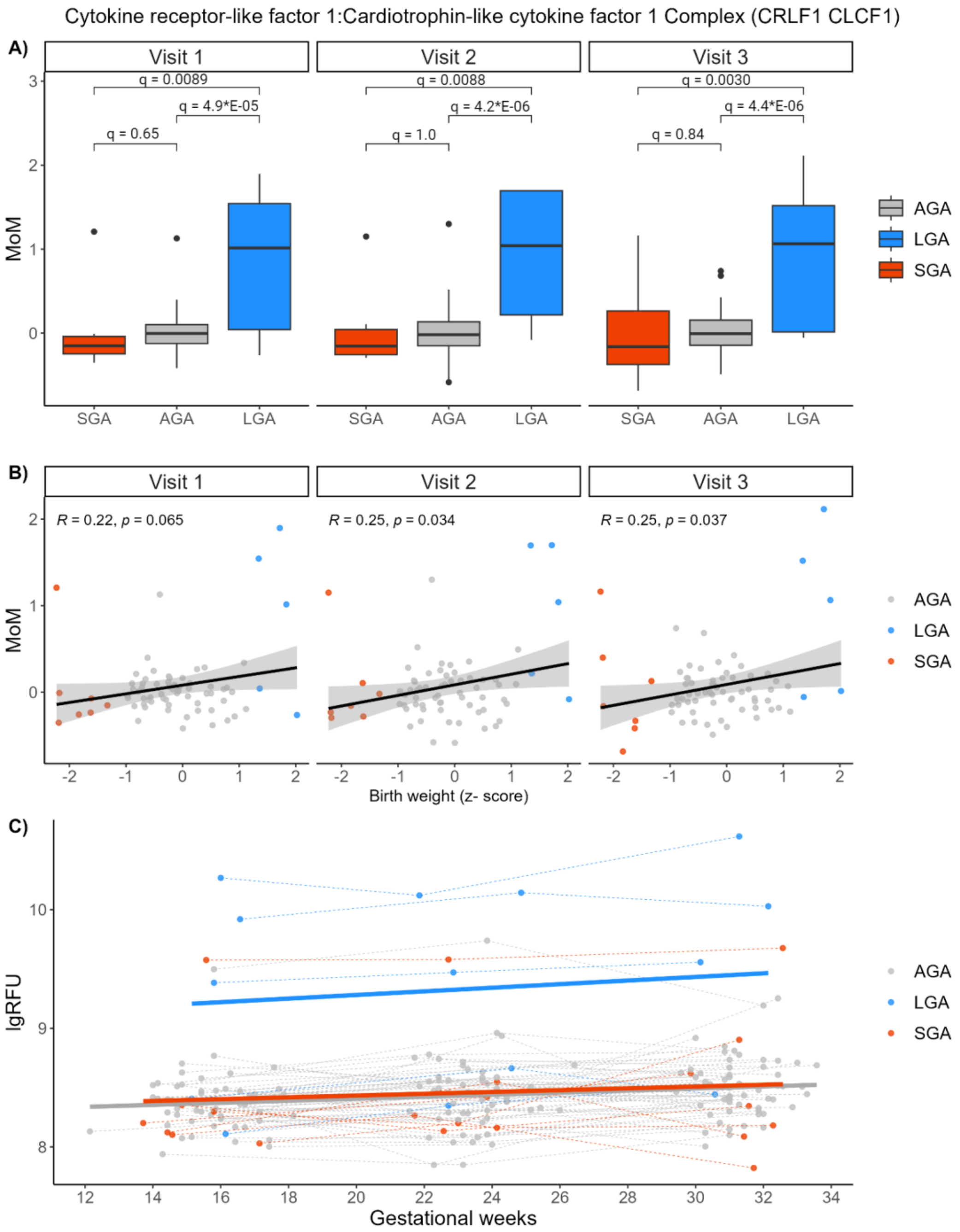

Supplementary *Figure S15.* *Protein expression patterns of Cytokine receptor-like factor 1:Cardiotrophin-like cytokine factor 1 Complex (CLF-1/CLC complex).* ***A****) Box plots displaying MoM values of CLF-1/CLC complex at visit 1, 2 and 3, with q-values from moderated t-tests between SGA, AGA and LGA groups.* ***B****) Pearson correlation between CLF-1/CLC complex MoM values and birth weight z-scores.* ***C****) Log RFU values of CLF-1/CLC complex across gestation. Mean curves within each birth weight groups are fitted a generalized additive models.*

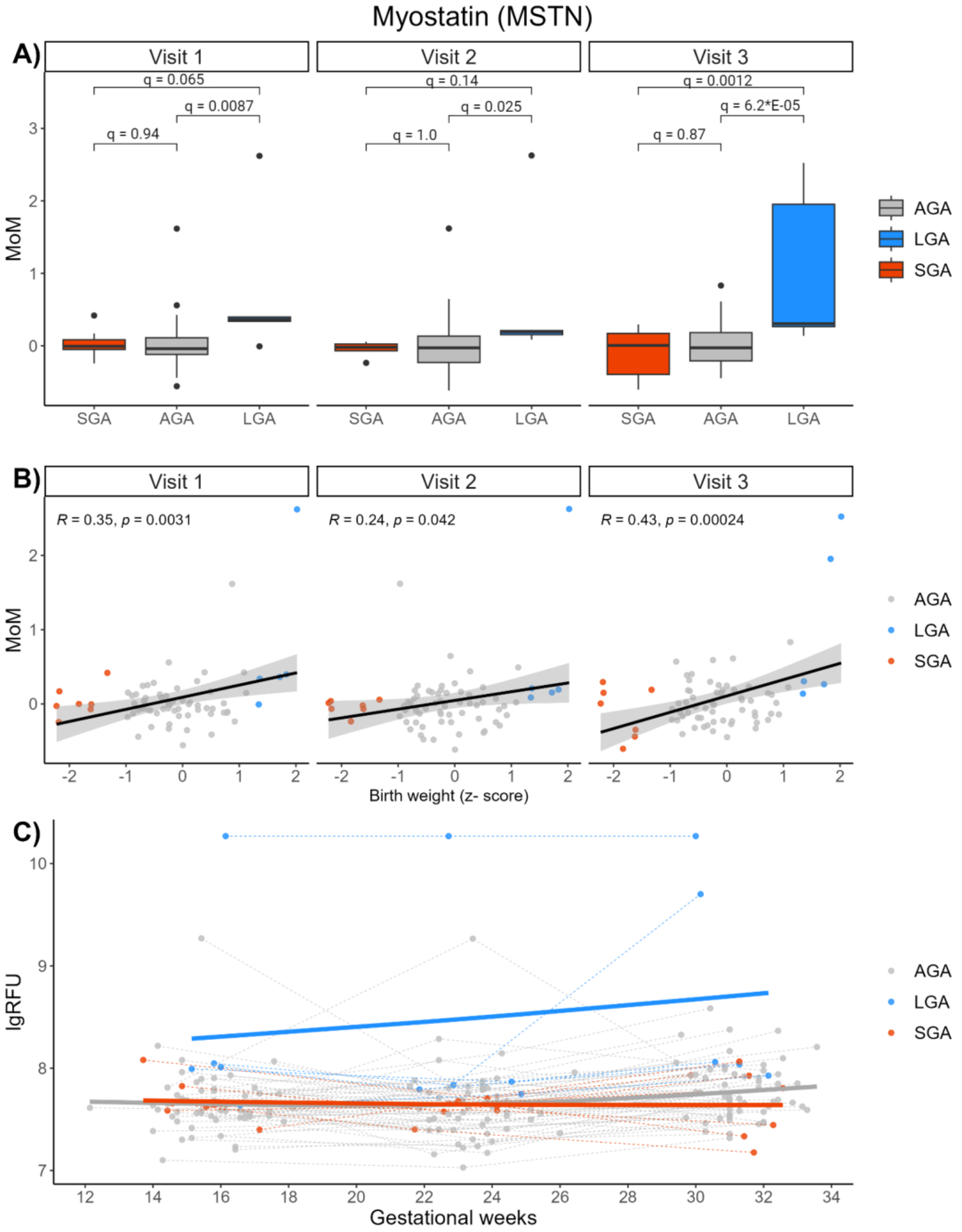

Supplementary ***Figure S16.*** *Protein expression patterns of Myostatin (MSTN).* ***A****) Box plots displaying MoM values of MSTN at visit 1, 2 and 3, with q-values from moderated t-tests between SGA, AGA and LGA groups.* ***B****) Pearson correlation between MSTN MoM values and birth weight z-scores.* ***C****) Log RFU values of MSTN across gestation. Mean curves within each birth weight groups are fitted a generalized additive models.*

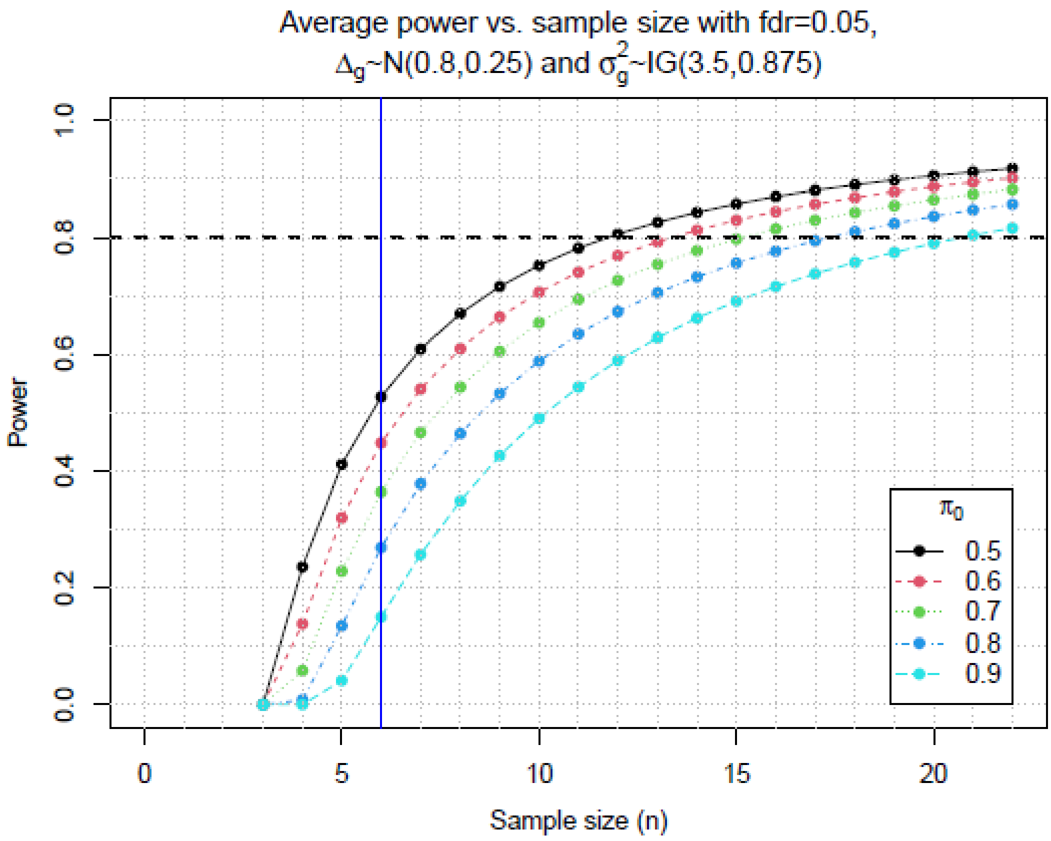

Supplementary *Figure S17.* *Power calculation with fixed sample size (x-axis), false discovery rate (FDR) and proportion of differentially abundant proteins (π_0) based on data from Tarca, Romero (1). With FDR = 0.05 and a sample size of six per group (indicated by the blue line) we expect <20% power if we assume that 10% of the proteins are differential (light blue graph) and <55% power if we assume that 50% of the proteins are differential (black graph). Calculations have been made with the ssize.fdr package in R (2).*

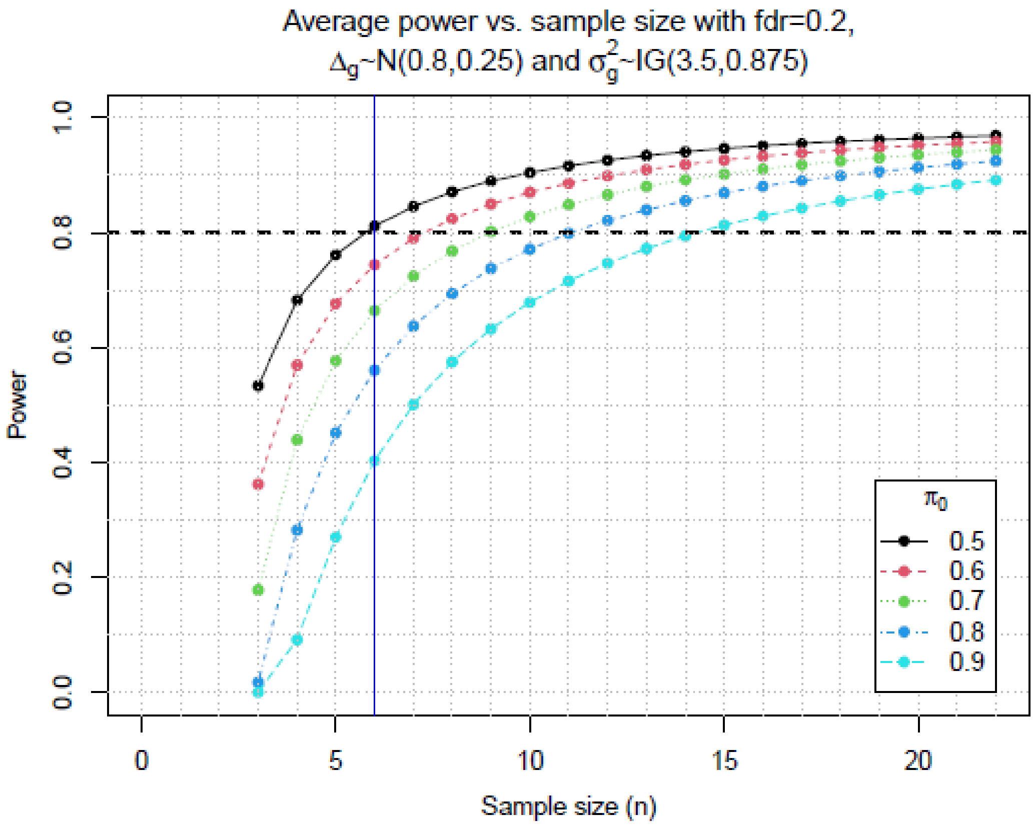

Supplementary *figure S18.* *Power calculation with fixed sample size (x-axis), false discovery rate (FDR) and proportion of differentially abundant proteins (π_0) based on data from Tarca, Romero (1). With FDR = 0.2 and a sample size of six per group (indicated by the blue line) we expect 40% power if we assume that 10% of the proteins are differential (light blue graph) and >80% power if we assume that 50% of the proteins are differential (black graph). Calculations have been made with the ssize.fdr package in R (2).*

References

1. Tarca AL, Romero R, Benshalom-Tirosh N, Than NG, Gudicha DW, Done B, et al. The prediction of early preeclampsia: Results from a longitudinal proteomics study. PloS one. 2019;14(6):e0217273.
2. Orr M, Liu P. Sample Size Estimation while Controlling False Discovery Rate for Microarray Experiments Using the ssize.fdr Package. R J. 2009;1.
